# Precision caffeine therapy for apnea of prematurity and circadian rhythms: New possibilities open up

**DOI:** 10.3389/fphar.2022.1053210

**Published:** 2022-12-01

**Authors:** Hao-Ran Dai, Hong-Li Guo, Ya-Hui Hu, Jing Xu, Xuan-Sheng Ding, Rui Cheng, Feng Chen

**Affiliations:** ^1^ Pharmaceutical Sciences Research Center, Department of Pharmacy, Children’s Hospital of Nanjing Medical University, Nanjing, China; ^2^ School of Basic Medicine and Clinical Pharmacy, China Pharmaceutical University, Nanjing, China; ^3^ Neonatal Intensive Care Unit, Children’s Hospital of Nanjing Medical University, Nanjing, China

**Keywords:** apnea of prematurity, caffeine, chronopharmacology, circadian rhythms, preterm infants

## Abstract

Caffeine is the globally consumed psychoactive substance and the drug of choice for the treatment of apnea of prematurity (AOP), but its therapeutic effects are highly variable among preterm infants. Many of the molecular underpinnings of the marked individual response have remained elusive yet. Interestingly, the significant association between *Clock* gene polymorphisms and the response to caffeine therapy offers an opportunity to advance our understanding of potential mechanistic pathways. In this review, we delineate the functions and mechanisms of human circadian rhythms. An up-to-date advance of the formation and ontogeny of human circadian rhythms during the perinatal period are concisely discussed. Specially, we summarize and discuss the characteristics of circadian rhythms in preterm infants. Second, we discuss the role of caffeine consumption on the circadian rhythms in animal models and human, especially in neonates and preterm infants. Finally, we postulate how circadian-based therapeutic initiatives could open new possibilities to promote precision caffeine therapy for the AOP management in preterm infants.

## 1 Introduction

Caffeine, one of the bioactive methylxanthines that exist in a variety of natural and processed foods and beverages, is the most frequently consumed psychoactive substance in the world (Gonzalez de Mejia and Ramirez-Mares, 2014; van Dam et al., 2020; Rodak et al., 2021). Studies have confirmed that ingested caffeine has profound effects on the function and health of various systems in the human body through the combination of several molecular mechanisms including the antagonism of adenosine receptors, inhibition of phosphodiesterase, and mobilization of intracellular calcium (Nehlig et al., 1992; Cappelletti et al., 2015; Rodak et al., 2021; Yang et al., 2021). Among these effects of caffeine, the most well-known are those on the central nervous system, such as the regulation of sleep-wake states, learning-memory functions, cognitive-behavioral performances, attention-alertness functions, and mood-consciousness states (Nehlig et al., 1992; Snel and Lorist, 2011; Spaeth et al., 2014; Urry and Landolt, 2015). Therefore, it is no surprise that many people are accustomed to taking caffeinated beverages or foods to combat sleep deprivation induced fatigue and circadian rhythm sleep disorder caused by shift work or rapid transmeridian travel (Landolt, 2015; Clark and Landolt, 2017; Arendt, 2018), while some other people intentionally avoid caffeine in their daily life so as not to interfere with regular sleep habits (Snel and Lorist, 2011).

On the other hand, the therapeutic use of caffeine is very common in clinical practice. Caffeine acts as a potent analgesic adjuvant and is often added to a variety of over-the-counter and prescription analgesics due to its anti-inflammatory and vasoconstrictive effects (Cappelletti et al., 2015; van Dam et al., 2020; Rodak et al., 2021). More strikingly, caffeine is the drug of choice for the treatment of apnea of prematurity (AOP) (Eichenwald et al., 2016; Kumar and Lipshultz, 2019; Moschino et al., 2020; Long et al., 2021) and becomes one of the most commonly prescribed medications in the neonatal intensive care unit (NICU) (Hsieh et al., 2014; Krzyżaniak et al., 2016), evidenced by its short-term and long-term efficacy and safety in reducing apnea, facilitating extubation, preventing bronchopulmonary dysplasia, ameliorating retinopathy of prematurity, reducing patent ductus arteriosus, and improving neurodevelopmental outcome that have been demonstrated in the Caffeine for Apnea of Prematurity (CAP) trial (Schmidt et al., 2006; Schmidt et al., 2007). Assuredly, compared with other methylxanthines, caffeine has higher therapeutic index, longer half-life, and better tolerability (Henderson-Smart and De Paoli, 2010; Henderson-Smart and Steer, 2010; Abdel-Hady et al., 2015). Inspiringly, caffeine has been clinically applied in the treatment of AOP for nearly 50 years, which has created a typical successful story in pediatrics (Kreutzer and Bassler, 2014; Dobson and Hunt, 2018; Williamson et al., 2021).

Recently, the association between caffeine and circadian rhythms has attracted widespread attentions (Landolt, 2015). Many intriguing phenomena occurred, and the underlying mechanisms have been tentatively investigated by several studies conducted in adults and animals (Spaeth et al., 2014), but we still know very little about the truth. Fortunately, however, our previous study revealed that the circadian rhythms in premature infants might play a sophisticated role in determining the efficacy of caffeine therapy (Guo et al., 2022). Therefore, it will be very interesting to summarize the current relevant studies to know about the progress of this research field.

To the best of our knowledge, there is no comprehensive summary of the most recent advances in the circadian rhythms in preterm infants and caffeine therapy. Thus, to fill this knowledge gap, in this review, we begin by introducing the coexistence of tough challenges and new insights in the current caffeine therapy for AOP. Then, our novel findings (Guo et al., 2022) push us to delineate the functions and mechanisms of human circadian rhythms first for better understanding the deep theoretical logic underlying those clinical phenotypes. As a key part of circadian development, an up-to-date knowledge of the formation and ontogeny of human circadian rhythms during the perinatal period are also concisely discussed. Undoubtedly, what attracts our attention the most is the research progress on the effects of caffeine on human circadian rhythms, especially for premature infants, and the progress on the sophisticated roles of circadian rhythms in the response to caffeine therapy for those babies with AOP. Therefore, based on the increasing evidence, a new possibility opens up in this area of research in light of the circadian rhythms.

## 2 Tough challenges and new findings in current caffeine therapy for AOP

To be honest, the tough challenges are always there for the current AOP therapy with caffeine. The optimal dose regimen, timing and duration of therapy, necessity of therapeutic drug monitoring, and variable clinical outcomes of caffeine in preterm infants remain controversial (Gentle et al., 2018; Davis, 2020; Saroha and Patel, 2020). Impressively, however, those problems related to the clinical use of caffeine in preterm infants have been widely concerned and discussed as the continuous deepening of research, especially as the application of several innovative research technologies, such as artificial intelligence, predictive modeling, and machine learning (Koch et al., 2017; Shirwaikar, 2018; Faramarzi et al., 2021; Dai et al., 2022). Interestingly, several novel findings in those studies provide valuable references for determining the optimal initial dose, tailoring the maintenance dose, enhancing clinical decision making, and then for promoting the achievement of consensus on those tough challenges (Abdel-Hady et al., 2015; Eichenwald, 2020; Moschino et al., 2020).

The clinical response bears the brunt. The most tough and urgent problem is the significant interindividual variability in response to caffeine therapy (He et al., 2021). It remains unclear why some preterm infants have well-controlled outcomes while others have not. To make matters worse, the frequent episodes of apnea among those lacking efficacy cannot be well controlled by solely increasing the dose of caffeine (Saroha and Patel, 2020).

Tentatively to explore the underlying factors that determine the interindividual response to caffeine therapy, a single-center and retrospective study was conducted by our team (He et al., 2021; Guo et al., 2022). In line with previous study (Saroha and Patel, 2020), the plasma concentration of caffeine could not explain the variable efficacy for preterm infants yet (He et al., 2021). Arguably, such highly variable response could not be explained either by the genetic polymorphisms of various genes encoding the metabolic enzymes and transporters (Guo et al., 2022). However, genetic polymorphisms involved in caffeine’s target receptors, directly and indirectly, and quite unexpectedly, in regulation of circadian rhythms were significantly associated with the variable response to caffeine therapy (Guo et al., 2022). Such novel finding bears good clinical significance and is inspirational for future studies to delve into the biological mechanisms.

## 3 The functions and mechanisms of human circadian rhythms

Due to the rotation of Earth, almost all life forms on the planet have evolved a biological timer to adapt to the daily changes in the environment (Du Pre et al., 2014; Dong et al., 2020; Jha et al., 2021). The endogenous biological clock is commonly called as the circadian (from Latin, meaning “about a day”) rhythms (Dong et al., 2020; Ruan et al., 2021). It is proven that the inherent period of the human pacemaker clock is close to 25 h in most people (Ohdo et al., 2019; Dong et al., 2020). However, because of the entrainment by environmental time signals, or so-called zeitgebers (from German, meaning “time givers”) (Bicker et al., 2020; Ruan et al., 2021), the inherited circadian pacemaker manifests itself in a 24-h pattern (Ohdo et al., 2019; Dong et al., 2020).

### 3.1 The functions of human circadian rhythms

Circadian rhythms regulate various behavioral, physiological, psychological, and endocrine functions in humans (Froy, 2007; Ribas-Latre and Eckel-Mahan, 2016; Allada and Bass, 2021; Kinouchi et al., 2021; Thosar and Shea, 2021; Zhang and Jain, 2021). One can imagine that circadian dysfunction would cause multiple negative impacts, both short term and long term, which lead to the increased susceptibility to many diseases, decreased quality of life, and even reduced life expectancy (Froy and Miskin, 2007; Jagannath et al., 2013; Roenneberg and Merrow, 2016; Valenzuela et al., 2016; Logan and McClung, 2019; Xu and Lu, 2019; Allada and Bass, 2021). Interestingly, the onsets and symptoms of many diseases, such as stroke, asthma, and depression, also display clear circadian characteristics (Jagannath et al., 2013; Hsieh et al., 2018; Cederroth et al., 2019; Dobrek, 2021; Ruan et al., 2021), which are called as the circadian pathology signs (Cederroth et al., 2019). Speaking of pharmacology, circadian rhythms affect the absorption, distribution, metabolism, and excretion (ADME) or called the pharmacokinetic processes as well as the efficacy and adverse effects of many drugs, which is well known as the chronopharmacology or chronotherapy (Dallmann et al., 2016; Ohdo et al., 2019; Dong et al., 2020; Dobrek, 2021; Nahmias and Androulakis, 2021). Given the importance of circadian rhythms, three researchers who discovered the basic of biological clock in studies of *Drosophila* were awarded the Nobel Prize in 2017 (Dobrek, 2021; Ruan et al., 2021).

### 3.2 The mechanisms of human circadian rhythms

**FIGURE 1:**
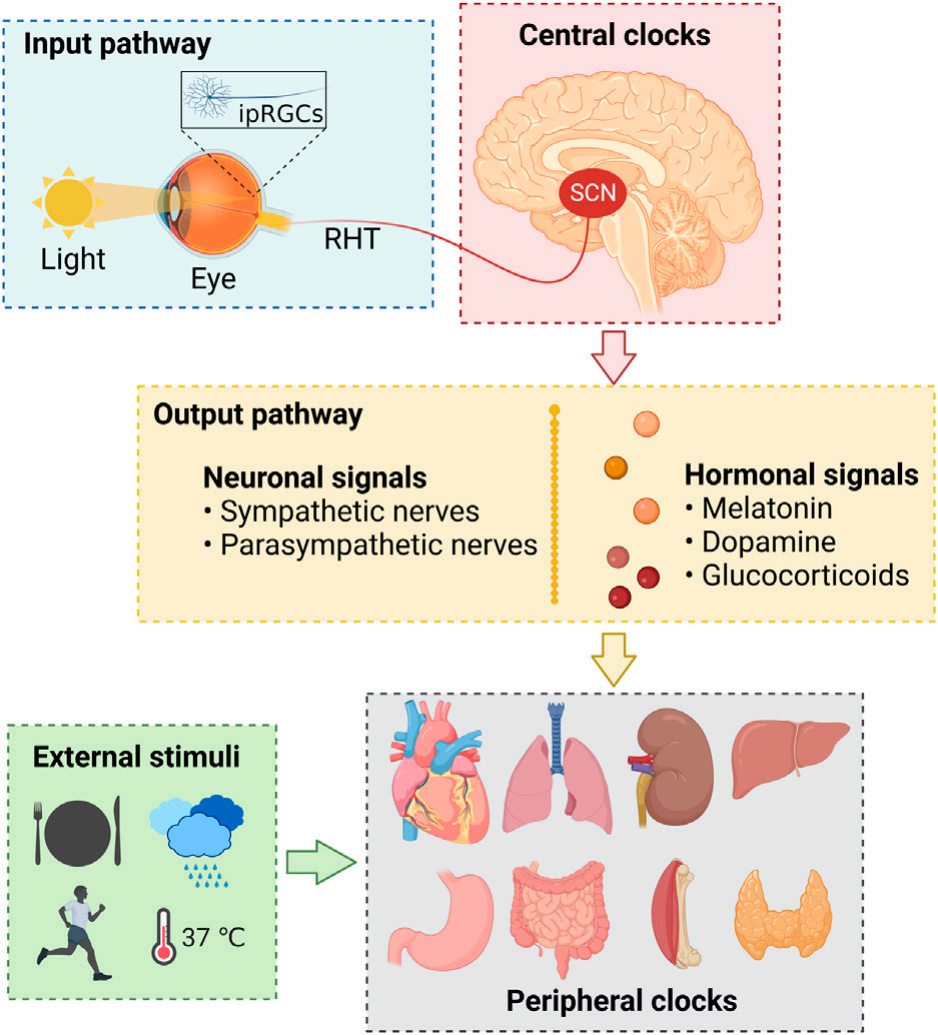
The physiological basis of human circadian rhythms. ipRGCs, intrinsically photosensitive retinal ganglion cells; RHT, retinohypothalamic tract; SCN, suprachiasmatic nuclei.

**FIGURE 2:**
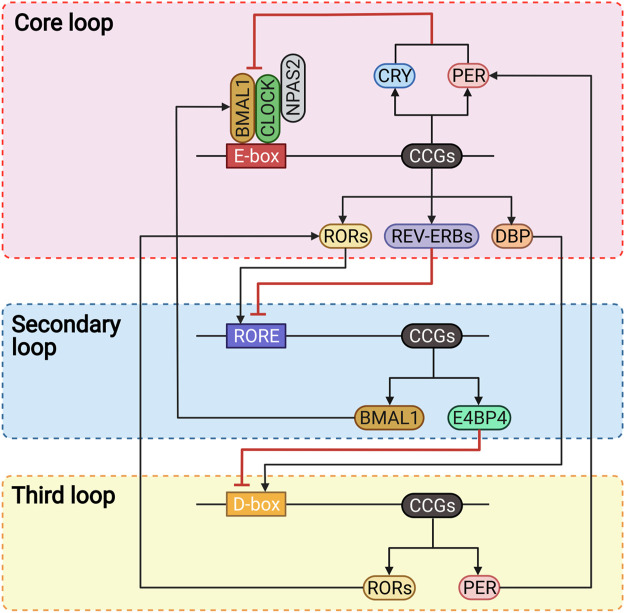
The molecular mechanism of human circadian rhythms. BMAL1, brain and muscle ARNT-like 1; CCGs, clock-controlled genes; CLOCK, circadian locomotor output cycles kaput; CRY, cryptochrome; DBP, D-box binding protein; E4BP4, E4 promoter-binding protein 4; NPAS2, neuronal PAS domain-containing protein 2; PER, period; RORE, ROR/REV-ERB response elements; RORs, retinoic acid receptor-related orphan receptors.

Back in the 1990s, the discovery of several circadian clock genes, such as *Clock*, *Bmal1*, *Per*, and *Cry* (Takahashi, 2004), proved that almost all human cells express these genes and have the capacity to generate circadian oscillations (Du Pre et al., 2014; Takahashi, 2017), which thwarted the previous neuro-centric view that the master clock is located only in the brain (Takahashi, 2017). As is generally believed and well understood, at the systemic level, the human circadian system consists of the inputs, circadian oscillators, and outputs (Figure 1) (Takahashi, 2017; Cederroth et al., 2019; Huang et al., 2020; Ruan et al., 2021), while at the cellular level, it consists of several cell-autonomous molecular oscillators that composed of three transcriptional-translational feedback loops that are widespread throughout the body (Figure 2) (Du Pre et al., 2014; Takahashi, 2017; Logan and McClung, 2019; Huang et al., 2020; Sumova and Cecmanova, 2020; Ruan et al., 2021).

#### 3.2.1 Physiological basis

The regulation and maintenance of human circadian rhythms depend on the synergy of the input pathways, central and peripheral clocks, and output pathways (Figure 1) (Huang et al., 2020). The input pathway senses and transmits the environmental rhythm signals to the central circadian clocks (Ruan et al., 2021), which act as the biological rhythm pacemaker to transmit the generated rhythm signals to the periphery through the output pathway (Du Pre et al., 2014; Jha et al., 2021), and then cooperate with the endogenous clock systems of peripheral tissues and organs to regulate the gene expression, cellular function, physiological activity, and metabolism of the body (Huang et al., 2020).

Light, the major input signal in the suprachiasmatic nuclei (SCN) of the circadian system, is perceived by the intrinsically photosensitive retinal ganglion cells (ipRGCs) (Zele et al., 2011), which express the photopigment melanopsin and are modulated by the rods and cones in the retina (Van Cruchten et al., 2017). Then, the ipRGCs generated and transmitted electric rhythm signals to the central clock system that located in the SCN of the hypothalamus through a neural pathway called the retinohypothalamic tract (RHT) (Logan and McClung, 2019; Dong et al., 2020; Jha et al., 2021).

The SCN is comprised of neurons that express the neuropeptide arginine vasopressin (AVP) and vasoactive intestinal polypeptide (VIP), which are essential for the circadian light transduction (Ono et al., 2021). The AVP and VIP neurons in the SCN master pacemaker are also regulated by the neurotransmitters released by the ipRGCs, such as excitatory glutamate and pituitary adenylate cyclase-activating polypeptide (PACAP) (Dong et al., 2020; Jha et al., 2021; Ruan et al., 2021). Subsequently, the SCN transmits such perceived rhythm information *via* neuronal and hormonal signals (Logan and McClung, 2019), and coordinates other oscillators in extra-SCN brain regions and peripheral tissues and organs, such as heart, lung, liver, and kidney (Takahashi, 2017; Huang et al., 2020).

It is worth mentioning that in addition to be regulated by the SCN master pacemaker, the peripheral clocks could also directly and SCN-independently receive external stimuli, such as food intake, exercise, temperature, and humidity (Figure 1) (Du Pre et al., 2014; Xu and Lu, 2019; Huang et al., 2020).

#### 3.2.2 Molecular mechanism

Three interlocked transcriptional feedback loops constitute the complex molecular clock networks at the cellular level (Figure 2) (Takahashi, 2017; Ruan et al., 2021). The core loop regulates human circadian rhythms with a period of approximately 24-h through a negative feedback mechanism (Huang et al., 2020; Allada and Bass, 2021). The circadian locomotor output cycles kaput (CLOCK) or neuronal PAS domain-containing protein 2 (NPAS2) forms heterodimers with the brain and muscle ARNT-like 1 (BMAL1) *via* binding to the E-box elements to regulate the transcription of clock-controlled genes (CCGs), including those encoding the period (PER) and cryptochrome (CRY) proteins (Takahashi, 2017; Logan and McClung, 2019; Dong et al., 2020). PER and CRY proteins accumulate in the cytoplasm in the morning (Ruan et al., 2021), then heterodimerize and translocate into the nucleus as negative regulators directly interact with CLOCK-BMAL1 to suppress their transcriptional activity in the late afternoon or evening (Takahashi, 2017; Xu and Lu, 2019). As the suppression progresses, PER and CRY proteins are gradually degraded *via* the ubiquitination through specific E3 ligase complexes and *via* the proteasome (Takahashi, 2017). At the same time, the transcription activity of CLOCK-BMAL1 is restored, and a new cycle will restart over the next morning (Ruan et al., 2021).

Besides, another two families of nuclear receptors, REV-ERBs and retinoic acid receptor-related orphan receptors (RORs), are also the direct targets of CLOCK-BMAL1 that stabilize the core loop, regulate the transcription in a distinct phase, and thus form the secondary or called the stabilization loop (Xu and Lu, 2019). The REV-ERBs inhibit the transcription of BMAL1 by competitively binding to the ROR/REV-ERB response elements (RORE) (Hsieh et al., 2018; Ruan et al., 2021). Conversely, the RORs are the positive regulators that bind to the RORE to promote the transcription of BMAL1 (Logan and McClung, 2019; Huang et al., 2020).

The third loop involves the proline- and acidic amino acid-rich basic leucine zipper (PAR-bZIP) factors, such as the D-box binding protein (DBP) and the repressor E4 promoter-binding protein 4 (E4BP4), which competitively bind to the D-box elements, and are driven by the core loop and stabilization loop, respectively (Takahashi, 2017; Ruan et al., 2021). DBP and E4BP4 contribute to circadian robustness by synergistically regulating the expression of RORs and PER proteins (Takahashi, 2017; Dong et al., 2020; Ruan et al., 2021).

Collectively, these three interactive feedback loops regulate the transcription and translation of CCGs by binding to the *cis*-elements, including E-box, RORE, and D-box, in their gene promoter and enhancer element regions (Dong et al., 2020; Ruan et al., 2021). In addition to these three transcriptional-translational feedback loops, several post-transcriptional and post-translational mechanisms, such as phosphorylation, acetylation, and ubiquitination of circadian proteins, also play important roles in regulating the circadian rhythms (Figure 2) (Takahashi, 2017; Xu and Lu, 2019; Huang et al., 2020).

## 4 The formation and development of human circadian rhythms

The physiological and molecular mechanisms of human circadian rhythms have been well described, but the formation and development during ontogenesis remain poorly understood (Astiz and Oster, 2020; Sumova and Cecmanova, 2020). Moreover, most studies were performed in rodents and non-human primates, which hinders our understanding of the developmental circadian physiology for humans (Rivkees, 2003; Sumova and Cecmanova, 2020). Nevertheless, the existing evidence reveals that the formation and development of circadian rhythms are the continuously morphological, structural, and functional maturation processes of tissues and organs with ontogenesis (Rivkees, 2003; Seron-Ferre et al., 2012).

### 4.1 The formation of circadian rhythms: Does fetus have circadian rhythms?

As early as 1975, a rat study (Deguchi, 1975) found, for the first time, that the mammalian fetal clock oscillators could be detected already at or before birth and be entrained by the mother. Subsequent studies have revealed that the fetus of rat, hamster, sheep, baboon, and other mammalians exhibited the circadian rhythms of metabolic activity (Reppert, 1992; Serón-Ferré et al., 1993; Mirmiran and Lunshof, 1996; Seron-Ferre et al., 2012) and the expressions of canonical clock genes (Seron-Ferre et al., 2007; Du Pre et al., 2014; Sumova and Cecmanova, 2020).

In human fetus, circadian rhythms in several physiological and endocrine functions, including heart rate (Lunshof et al., 1998), breathing patterns (Patrick and Challis, 1980), limb movements (Einspieler et al., 2021), sleep-wake states (Peirano et al., 2003; Bennet et al., 2018), and hormone levels (Serón-Ferré et al., 2001a) have been detected at different stages of pregnancy (Seron-Ferre et al., 2007; Du Pre et al., 2014; Wong et al., 2022). Impressively, Frigato et al. (2009), first observed the rhythmic expression of clock genes such as *Per2* in the HTR-8/SVneo cells derived from human extravillous trophoblast. As part of a series of important discoveries, Perez et al. (2015) went on to find the rhythmic expression of various circadian genes, including *Clock*, *Bmal1*, *Per2*, and *Cry1* in human full-term placenta.

It is incredible that no obvious circadian rhythms were found in the anencephalic fetus despite an intact maternal circadian rhythms were detected through the 24-h period fetal heart rate monitoring for anencephaly (Mirmiran and Lunshof, 1996), which demonstrated that the fetal brain, especially in the SCN, is required for the generation of fetal circadian rhythms (Mirmiran and Lunshof, 1996). It is still unclear when the fetal SCN clock first appeared morphologically, yet through the *in vitro* autoradiography by ^125^I-labeled melatonin, the SCN is apparent as discrete nuclei in the human fetus and already has melatonin receptors at 18 weeks of gestational age (GA) (Reppert et al., 1988). Besides, it has been demonstrated that the VIP and AVP neurons were first observed at 31 weeks of GA in the ventrolateral part of the fetal SCN (Swaab et al., 1990; Swaab et al., 1994). Therefore, it is currently recognized that the circadian rhythms in humans are formed and developed during the perinatal period (Rivkees, 1997; Sumova and Cecmanova, 2020), while the components of the circadian system like the SCN are established and functional early in human fetus (Serón-Ferré et al., 1993).

### 4.2 Prenatal circadian rhythms: Complex interaction of maternal, placental, and fetal circadian systems

Pregnancy presents an unusual circadian physiology pattern in which the fetal circadian system is completely embodied within that of the mother (Mark et al., 2017), and the two systems are connected by the placenta and interact with each other through this interface (Mark et al., 2017; Astiz and Oster, 2020; Bates and Herzog, 2020). Generally, placenta is responsible for the bidirectional transference of nutrients, hormones, metabolites, and gases (*i.e.,* oxygen and carbon dioxide) between the mother and fetus (Seron-Ferre et al., 2012; Valenzuela et al., 2015; Astiz and Oster, 2020). Meanwhile, the placenta conveys the maternal circadian timing cues, such as physical activity, feeding behavior, temperature, heart rate, blood pressure, and hormonal levels, to the fetus (Serón-Ferré et al., 2001a; Seron-Ferre et al., 2012). In particular, multiple hormones produced by the mother, including melatonin, dopamine, glucocorticoids, estrogen, and progesterone, have profound effects on the development and entrainment of the fetal circadian rhythms (Mirmiran and Lunshof, 1996; Rivkees, 1997; Seron-Ferre et al., 2007; Mark et al., 2017). In addition, hormones such as human chorionic gonadotropin (hCG), secreted by the placenta, also exhibit obvious circadian characteristics (Waddell et al., 2012; Mark et al., 2017; Bates and Herzog, 2020). It will be very interesting to know how those hormones affect the formation of the fetal circadian rhythms.

#### 4.2.1 Melatonin

Melatonin, known as the hormone of night (Seron-Ferre et al., 2007), can be secreted by various organs, including the pineal gland, ovary, and placenta (Itoh et al., 1999; Lanoix et al., 2008; Reiter et al., 2013; Reiter et al., 2014). However, melatonin is not synthesized by the fetal pineal gland or other organs (Mark et al., 2017), so the fetus must rely on the maternal melatonin for photoperiodic information since the unaltered melatonin readily crosses the placenta and distributes to the fetal tissues (Waddell et al., 2012; Reiter et al., 2014; Valenzuela et al., 2015). During normal human gestation, the nighttime peak melatonin level decreases slightly between the first and second trimesters, but begins to increase after 24 weeks, then increases to significantly high levels after 32 weeks, thereafter reaches its peak at the end of pregnancy, and finally returns to the pre-pregnancy level on the day after parturition (Nakamura et al., 2001; Mark et al., 2017). Late in human pregnancy, uterine contractions become intensest during the night as melatonin levels are at their highest (Nakamura et al., 2001), and the peak melatonin at the end of pregnancy is thought to promote uterine contractions that necessary for delivery (McCarthy et al., 2019).

Studies have demonstrated that the onset of human term delivery is more commonly between the late night and the early morning (Glattre and Bjerkedal, 1983; Cooperstock et al., 1987). Similar circadian characteristics of delivery were also observed in preterm labors after 28 weeks of GA (Lindow et al., 2000; Iams et al., 2002), but not in those before 28 weeks (Vatish et al., 2010), which might be explained by the immaturity of fetal circadian system or other pathological factors that bypass the physiological circadian process of labor (Vatish et al., 2010). Interestingly, studies revealed that the elevated nocturnal levels of melatonin synergized with oxytocin to trigger and maintain the uterine contractions during labor and that melatonin sensitized the human uterine to oxytocin (Reiter et al., 2014; Carlomagno et al., 2018; Chuffa et al., 2019). Consistently, women who engage in shift work during pregnancy have an increased incidence of spontaneous miscarriages, preterm deliveries, and low birth weight infants (Zhu et al., 2004; Croteau et al., 2006). Disruptions of the melatonin rhythms due to the shift work might be responsible for these adverse pregnancy outcomes (Reiter et al., 2014). In addition, as a free radical scavenger and an antioxidant, melatonin plays an important role in protecting the fetus and placenta from oxidative stress to promote the embryonic development and to treat the preeclampsia, intrauterine growth restriction, and the undernourished pregnancy (Reiter et al., 2014; Valenzuela et al., 2015; Rodrigues Helmo et al., 2018; Chuffa et al., 2019).

#### 4.2.2 Dopamine

As the antiphase and functionally antagonistic of melatonin, dopamine has been proposed as a “light-phase” entrainment signal of the circadian systems (Iuvone and Gan, 1995; Astiz and Oster, 2020). Plasma dopamine levels in humans peak around the waking time (about 08:00) and drop to a nadir in the middle of sleep (about 03:00) (Sowers and Vlachakis, 1984). Increased dopamine concentrations were detected in women’s amniotic fluid between the second and third trimesters, and were significantly higher than those in maternal and fetal plasma (Peleg et al., 1986), because dopamine could freely cross through the placenta into the fetal circulatory system (Watanabe et al., 1990). Furthermore, D1-dopamine receptors could be detected in the fetal SCN as early as 22 weeks of GA (Rivkees and Lachowicz, 1997). However, it remains unknown when and how the maternal dopamine entrains the circadian rhythms in fetus during the pregnancy (Bates and Herzog, 2020).

#### 4.2.3 Glucocorticoids

Cortisol, the glucocorticoid stress hormone, is regulated by the circadian of the hypothalamic-pituitary-adrenal (HPA) axis (Mark et al., 2017; Oster et al., 2017; McCarthy et al., 2019). During gestation, cortisol levels in maternal plasma peak in the early morning (from 07:30 to 08:30) and drop to a nadir at night (from 18:30 to 01:30) (Patrick et al., 1980). The maternal plasma cortisol levels increase progressively between 11 and 22 weeks of GA and then stay high until the initiation of delivery (Patrick et al., 1980; Carr et al., 1981). Such elevated maternal cortisol is critical for fetal tissue development, especially the maturation of the brain and lung (Matthews et al., 2004), and helpful for dampening the maternal stress signals to protect the fetus (McCarthy et al., 2019). Conversely, excessive cortisol level is detrimental for the fetal development that delaying the fetal and placental growth and increasing the risk of behavioral and mental disorders later in life (Busada and Cidlowski, 2017; Van den Bergh et al., 2020).

The placental glucocorticoid barrier regulates the glucocorticoids’ passage from the mother to the fetus *via* the enzyme 11β-hydroxysteroid dehydrogenase type 2 (11β-HSD2) that converts the biologically active glucocorticoids (*i.e.*, cortisol and corticosterone) to their inactive forms (*i.e.*, cortisone and 11-dehydrocorticosterone) (Edwards et al., 1996; Burton and Waddell, 1999). In humans, the glucocorticoids passage from the maternal to fetal circulation is gradually reduced due to the increasing placental 11β-HSD2 expression with advancing gestation (Burton and Waddell, 1999; McTernan et al., 2001). Impressively, glucocorticoid receptors have been identified in the fetal circulation, and maternal glucocorticoids could entrain fetal circadian rhythms through binding to these receptors (Bates and Herzog, 2020). Moreover, studies have found that the suppression of maternal adrenal function with glucocorticoid treatment resulted in a temporary disappearance of the fetal heart rate, breathing, and limb movement rhythms (Verdurmen et al., 2013). Interestingly, these inhibitory effects were dependent on the GA when the glucocorticoid therapy was started and disappeared with the restoration of the maternal HPX axis (Mulder et al., 2004), indicating the fetal rhythms depended on the maternal adrenal functions (Koenen et al., 2005).

#### 4.2.4 Sex hormones

The effects of sex hormones on the entrainments of fetal circadian rhythms are still under investigation. Estrogen and progesterone are two sex hormones that are essential for the successful pregnancy (Mark et al., 2017). During the first 3 months of pregnancy, estrogen and progesterone are synthesized and secreted by the ovary. After that, the placenta replaces the corpus luteum to secrete these two hormones, and estrogen is also produced by the uterus (McCarthy et al., 2019). The levels of estrogen and progesterone increase steadily over the human gestation due to an increased secretion from the ovary and placenta (Mark et al., 2017). During gestation, estrogen levels in maternal plasma peak in the morning and become lowest at midnight (Patrick et al., 1979; Challis et al., 1980), whereas a significant antiphase oscillation of the estrogen occur in the progesterone levels (Junkermann et al., 1982; Magiakou et al., 1996), which might be regulated by the circadian of placental glucocorticoids (Serón-Ferré et al., 1993).

Estrogen promotes the synthesis of progesterone (Babischkin et al., 1997), which regulates maternal immunity to facilitate implantation (Hardy et al., 2006), maintains uterine quiescence during pregnancy (Peters et al., 2017), and causes myometrial contractions to trigger labor at the end of pregnancy (Brown et al., 2004). Interestingly, these two hormones were found to inhibit the expression of 11β-HSD2 in human placental extracts, which possibly increased the transport of glucocorticoids from the mother to the fetus (Sun et al., 1998), thereby indirectly influencing the fetal circadian rhythms.

Collectively, much less is known about other rhythmic signals such as leptin, placental lactogen, prolactin, or hCG that generated by the mother or placenta on the development and entrainment of fetal circadian rhythms (Astiz and Oster, 2020; Bates and Herzog, 2020). Because the interactions among maternal, placental, and fetal circadian systems are critical to the establishment, maintenance, and success of pregnancy, and the interactions also affect the growth, development, and even postpartum life of the fetus (Mark et al., 2017; Bates and Herzog, 2020), further studies are still needed to elucidate the complex interactions among the three circadian systems and to bridge the above knowledge gaps.

### 4.3 Postnatal circadian rhythms: Progressive maturation along with ontogenesis

After birth, neonates immediately begin to establish their own physical and physiological independence while losing the protect of the maternal-placental barrier (Joseph et al., 2015; Wong et al., 2022). From now on, the ontogenesis of the newborn begins to be greatly affected by the external environment (Brooks and Canal, 2013; Hazelhoff et al., 2021). Increasing evidence indicates that human postnatal circadian rhythms gradually mature along with the ontogenesis (Rivkees and Hao, 2000; Rivkees, 2007; Bueno and Menna-Barreto, 2016), in which the external environment, especially the light, plays an important role in the development and maturation (Mirmiran and Ariagno, 2000; Nishihara et al., 2002; Challet, 2007). Particularly, it should be pointed out that, in early infancy, the maternal entrainment factors and maternal-fetal interactions retained during pregnancy are more important than the external environment (Löhr and Siegmund, 1999; Rivkees, 2001; Nishihara et al., 2002; Sumova et al., 2012).

#### 4.3.1 Maternal effects

The first thing to be discussed is the role of hormones. During the first few weeks of life, circadian rhythms in human neonates occur as the retentions of the maternal influence *in utero*, but the endogenous rhythms appear only later (Rivkees, 1997; Rivkees and Hao, 2000; Brooks and Canal, 2013). For example, an antiphase oscillation of maternal cortisol circadian rhythms (*i.e.*, the peak of cortisol levels occurred between 12:00 and 16:00) was found in the umbilical artery but not the umbilical vein of the term fetus (Serón-Ferré et al., 2001b), which reflects the activation of the intrinsic fetal HPA axis in response to the falling maternal transport of glucocorticoids during the nadir of the maternal rhythms (Mark et al., 2017). Moreover, the neonatal salivary cortisol levels were higher at night than in the morning within the first 8 weeks of postnatal age (PNA) (Iwata et al., 2013; Kinoshita et al., 2016), which were in consonance with the fetal cortisol rhythms (Serón-Ferré et al., 2001b), reflecting the preservation of fetal adrenal rhythms.

Neonates begin to exhibit the circadian salivary cortisol rhythms analogous to that of adults (*i.e.*, higher cortisol levels in the morning than at night) until 2–3 months of PNA (Price et al., 1983; Spangler, 1991; Mantagos et al., 1998; Joseph et al., 2015). However, an adult-type salivary cortisol circadian of term infants appears to be established actually at 1 month and remains stable throughout the first year of life (Ivars et al., 2015). All in all, these studies prove that the fetal cortisol circadian rhythms are preserved in the first few weeks of life, until the adult-type circadian rhythms are established.

The rhythm of melatonin is another example. (Muñoz-Hoyos et al., 1992; Muñoz-Hoyos et al., 1998) found that the adult-type circadian melatonin rhythms occurred in both the umbilical artery and vein for neonates at birth, which depended on the maternal melatonin crossing the placenta, as melatonin levels in the umbilical artery are positively correlated to those in the maternal serum and a similar correlation between the maternal and neonatal melatonin levels in the first voided urine after delivery (Kivelä et al., 1990). Besides, although the increasing amounts of melatonin and its metabolite 6-sulfatoxymelatonin were detected in the urine of the term neonates during the first week of life (Kivelä et al., 1990; Muñoz-Hoyos et al., 1993), the stable circadian melatonin rhythms were not developed until approximately 9–12 weeks of PNA (Attanasio et al., 1986; Kennaway et al., 1992; Kennaway et al., 1996; Joseph et al., 2015).

The second thing to be discussed is the maternal care, primarily maternal feeding, but it is still the roles of hormones in nature (Löhr and Siegmund, 1999; Nishihara et al., 2002; Park et al., 2020). Various hormones in breast milk, such as glucocorticoids and melatonin, can be absorbed and transferred into the neonatal circulation through the gastrointestinal tract (Arslanoglu et al., 2012; Wong et al., 2022). Interestingly, the cortisol and cortisone concentrations in breast milk follow the circadian of maternal HPA axis activity (van der Voorn et al., 2016; Italianer et al., 2020). Moreover, the cortisone rhythm in human breast milk at 1 month postpartum was associated with the nighttime sleep states of newborns at the age of 3 months (Toorop et al., 2020). Similarly, studies have also demonstrated the presence of pronounced circadian melatonin rhythms in the maternal breast milk (Illnerová et al., 1993; Katzer et al., 2016), which might contribute to the synchronization of postnatal circadian rhythms for neonates and their mothers.

One more thing needs to be pointed out is that, in addition to the maternal influence on the neonatal circadian rhythms, the maternal circadian rhythms are in turn affected by the development of the neonatal circadian rhythms (Nishihara and Horiuchi, 1998; Nishihara et al., 2000; Nishihara et al., 2002). For example, the ultradian rhythms (*i.e.*, rhythms with period lengths much less than 24 h) (Rivkees, 1997) of rest-activity states were already detected as early as the third week of life for term infants, and the amplitude of this rhythm gradually increased from the 6th to 12th week, then formed circadian rhythms with a 24-h monophasic pattern (Nishihara et al., 2002). During this period, as neonates develop their own circadian rest-activity rhythms, the mothers’ rhythms would inevitably be affected by their interrupted sleep at night to take care of their babies (Nishihara and Horiuchi, 1998; Nishihara et al., 2000).

#### 4.3.2 Environmental effects

In the late postnatal period, environmental time cues replace the maternal effects and begin to play a critical role in the development of neonatal circadian rhythms (Rivkees, 1997; Rivkees, 2004; Brooks and Canal, 2013). Light is the most dominant zeitgeber (Löhr and Siegmund, 1999; Challet, 2007; Wong et al., 2022), so the importance of light cannot be overemphasized. The light entrainments are functionally affected by the maturity of the eyes, RHT, and SCN (Brooks and Canal, 2013; Hazelhoff et al., 2021).

For term infants, the structural development of the eyes occurs as early as *in utero*, with the first structure of the eyes beginning to form at 17 days of GA (Van Cruchten et al., 2017), while the development of pupil starts approximately at 17 weeks of GA (Hazelhoff et al., 2021), and thereafter the pupillary light reflex already present at 34 weeks of GA (Robinson and Fielder, 1990). As the sole photoreceptive area in humans (Brooks and Canal, 2013), major classes of photoreceptors in the retina including the ipRGCs, rods, cones, and melanopsin all emerge and develop in the first trimester (Van Cruchten et al., 2017; Hazelhoff et al., 2021).

Covering the eyes of term neonates during the phototherapy for neonatal hyperbilirubinemia would result in significantly increased plasma melatonin levels during the first 72 h of life, indicating the sensitivity of the neonatal pineal glands to the changes of environmental illumination and the functional maturation of the neonatal eyes in transmitting the ambient light cues (Jaldo-Alba et al., 1993). However, it remains unclear when human ipRGCs transmit the light cues to the SCN, but the melanopsin-dependent ipRGCs in mice could provide light signals to the SCN already on the day of birth (Sekaran et al., 2005), and even earlier in late gestation before birth (Rao et al., 2013).

Honestly, only several studies reported the developmental process of human RHT and SCN. RHT has been identified in neonates at 36 weeks of GA (Rivkees, 2004; Rivkees, 2007). On the other hand, it has been found that the SCN of baboons born at term was already responsive to light and could be entrained by the low-intensity (200 lux) lighting (Rivkees et al., 1997). Interestingly, the SCN in preterm baboons functionally responded to light from a stage that was equivalent to 24 weeks of GA for human infants (Hao and Rivkees, 1999). Theoretically, the ambient light signals might be projected from the ipRGCs on retina to the SCN *via* the RHT at least after birth for term neonates (Hazelhoff et al., 2021). Further maturations of the human SCN continues after birth (Rivkees, 2004; Rivkees, 2007).

The numbers of AVP neurons and total neurons in the SCN of term neonates at birth are only 13% and 20% of those in adults, respectively (Swaab et al., 1990). After birth, these nerve cells increase rapidly to a peak at 1–2 years of age, then decrease gradually to the adult levels (Swaab, 1995). However, the development of VIP neurons in the SCN is slower and does not reach the adult levels until about 3 years of age (Swaab et al., 1994). Interestingly, there is a clear sex difference (*i.e.,* 2-fold higher in males than that in females) in the number of VIP neurons after 10 years of age (Swaab et al., 1994), which suggested a possibility that the SCN involves not only in the timing of circadian rhythms, but also in the temporal organization of sexually dimorphic reproductive functions (Swaab, 1995; Hofman, 1997).

The impact of light on the clock gene expression is also a research progress worthy of special attention. The light affects the expression of clock genes, such as *Per1*, *Per2*, and *Cry1*, in the SCN of rodents at different developmental stages after birth (Kováciková et al., 2005; Ciarleglio et al., 2011). Moreover, it is the cycled light rather than the constant light that promotes the development of their biological clocks (Abraham et al., 2006; Ohta et al., 2006; Bode et al., 2011). Impressively, human neonates, especially the preterm neonates who exposed to cycled light would have better weight gains (Mann et al., 1986; Brandon et al., 2002; Vasquez-Ruiz et al., 2014; Brandon et al., 2017), less crying and fussing behaviors (Guyer et al., 2012), less hospital stay (Vasquez-Ruiz et al., 2014; Brandon et al., 2017), earlier rest-activity rhythms (Rivkees, 2004; Rivkees et al., 2004), longer nighttime sleep duration (Guyer et al., 2015), and even more robust salivary melatonin rhythms (Vasquez-Ruiz et al., 2014) compared to those exposed to continuous light or darkness. Systematic reviews also witnessed the beneficial effects of cycled light over continuous bright light or darkness for preterm neonates (Morag and Ohlsson, 2016; Liao et al., 2018). Therefore, as early as the 1990s, the guidelines for perinatal care that proposed by the American College of Obstetricians and Gynecologists and American Academy of Pediatrics were recommended to introduce a regular day-night cycled light into the NICU and neonatal nursery (Mirmiran et al., 2003a; Guyer et al., 2015).

Besides the light cues, studies have pointed out that the environmental noise disrupted the neurodevelopment of newborns and thus affected the development of their circadian rhythms (Wachman and Lahav, 2011; Kuhn et al., 2013). However, music therapy did improve the heart rate, breathing, and sleep of newborns (Arnon et al., 2006; Loewy et al., 2013), which might exert a positive impact on the well-being and quality of life for neonates, especially for preterm infants in the NICU (Yue et al., 2021). Other environmental factors, such as ambient temperature (Tourneux et al., 2008), comforting touch (Smith et al., 2014), remodeling mattress (Deiriggi, 1990; Visscher et al., 2015), and nursing measures (Collins et al., 2015; Lan et al., 2018) were also found to affect the neonatal rhythms of several physiological parameters, but their roles on the development of circadian rhythms in neonates have not been extensively studied yet (Liao et al., 2018; Gogou et al., 2019).

## 5 The characteristics of circadian rhythms in preterm infants

Preterm birth is defined as a live birth that occurs before 37 completed weeks of GA (Walani, 2020), which causes the fetus to detach prematurely from the natural protective environment of the uterus (Vohr, 2013; Hazelhoff et al., 2021) and puts an early end of fetal development in the uterus, especially for the brain and lung, which are critical to the neonates’ survival after birth (Saigal and Doyle, 2008). Preterm infants have an increased risk of short-term and long-term morbidities (Deng et al., 2021), like the neurological and respiratory conditions (Vogel et al., 2018). Unfortunately, those babies continue to contribute disproportionately to neonatal mortality and even the childhood morbidity, which puts a heavy burden on health resources (Saigal and Doyle, 2008; Vohr, 2013).

Impressively, circadian rhythms in premature infants primarily occur as ultradian or irregular rhythms (Mirmiran et al., 2003a; Rivkees, 2007; Koch et al., 2021). It is hypothesized that the rhythms in preterm neonates appeared to be closely related to their GA (Begum et al., 2006; Darnall et al., 2006), due to the development of the fetal brain is related to the stages of pregnancy (Andescavage et al., 2017). On the other hand, the continuous active brain maturation occurs after birth (Matthews et al., 2018), so their endogenously-driven rhythms also change with the postmenstrual age (PMA) (Mirmiran et al., 2003a; Darnall et al., 2006). However, due to the remarkable heterogeneity in terms of methodological designs, the characteristics of the circadian rhythms in preterm infants have not been consistently described, and some studies have even found conflicting results (Mirmiran et al., 2003a). For comprehensively and precisely understanding the circadian rhythms in preterm infants, relevant advances are summarized in Tables 1–4 and discussed as follows:

**TABLE 1:** Studies about the sleep-wake rhythms in preterm infants.

Studies	Subjects	Methods of evaluation	Main findings
Guyon et al. (2022)	12 preterm infants (GA: 35.1 ± 2.1 weeks) *vs.* 21 term infants (GA: 39.8 ± 0.8 weeks)	Polysomnography	• Preterm *vs.* term infants: TST↓, AS↓, QS↑, arousal in AS↓, arousal in QS↑
			• With advancing PMA for preterm infants: TST and SE during day sleep↓, TST and SE during night sleep↑, AS↓, QS↑, arousal in AS↑, arousal in QS↓
Koch et al. (2021)	65 preterm infants (GA: 30.8 ± 2.1 weeks)	Video recordings	• Preterm infants spend about 43% of the time in AS, 38% in awake, and 19% in QS during the first 5 days of life
			• Sleep cycle durations of preterm infants range from 16 to 23 min with the average of 19 min
Georgoulas et al. (2021)	175 preterm and term infants (GA: 28–40 weeks)	Direct behavioral observations; EEG	• Preterm *vs.* term infants: AS↑, IS↑, QS↓, awake↓
			• With advancing PMA for preterm infants: AS↓, IS↓, QS↑, awake↑
Park et al. (2020)	94 preterm infants (GA: 26.2 ± 1.4 weeks)	Digitized waveforms	• With advancing PMA for preterm infants: AS↓, QS↑, waking states↑
			• Delayed feeding progression leads to delayed sleep-wake state development
Cailleau et al. (2020)	10 preterm infants (GA: 27–37 weeks) *vs.* 5 term infants (GA: 39–40 weeks)	Video recordings	• Preterm *vs.* term infants: QS↓
			• With advancing PMA for preterm infants: QS↑
Lan et al. (2019)	30 preterm infants (GA: 31.17 ± 2.6 weeks)	Actigraphy	• Sleep-wake patterns of preterm infants are associated with the gender, illness severity, PMA, and body weight
			• Preterm infants’ TST and percentage of sleep time are longer at night than during the day
			• With advancing PMA for preterm infants: TST↓, SE↓, percentage of sleep time↓, frequency of sleep and wake bouts↑
Cremer et al. (2016)	38 preterm infants (GA: 29.0 ± 2.6 weeks)	Video recordings	• Preterm infants with higher GA have longer awake times
			• Preterm boys have shorter awake times than girls
Bueno and Menna-Barreto, (2016)	19 preterm infants (GA: 28–36 weeks)	Actigraphy; Sleep and feeding diaries by the nurse	• Preterm infants exhibit the feeding-related 3-h period ultradian activity-rest rhythms after birth
			• Daily pattern circadian rhythms were observed for most preterm infants since 35 weeks of PMA
Guyer et al. (2015)	34 preterm infants (GA: 30.0 ± 1.8 weeks) *vs.* 21 term infants (GA: 39.7 ± 1.3 weeks)	Actigraphy; Parental sleep diaries	• Preterm *vs.* term infants: TST↑, LSP↑, nighttime sleep↑, nighttime activity↓
			• With advancing PMA for preterm infants: TST↓, LSP↑, nighttime sleep↑, daytime sleep↓, activity at daytime↑, activity at nighttime↑
Dorn et al. (2014)	60 preterm infants (GA: 30.0 ± 10.8 weeks)	Actigraphy	• Preterm infants primarily exhibit the 4-h period ultradian activity rhythms, with the most time in the low activity patterns
			• With advancing PMA for preterm infants: SE↑, activity frequencies↓, low activity patterns↑, middle and high activity patterns↓
Palmu et al. (2013)	12 preterm infants (GA: 24.7–30.3 weeks)	Polysomnography	• Only few premature infants exhibit about 20–50 min period ultradian sleep-wake rhythms due to the unstable respiratory states
			• Preterm infants have frequent transitions of sleep stages, spend most of time in AS, and the proportion is correlated with PMA
Lee et al. (2010)	35 preterm infants (GA: 24.9–31.9 weeks)	aEEG recordings	• The sleep-wake cycling is more prominent in preterm infants with higher PNA at 34–36 weeks PMA
			• The appearance of sleep-wake cycling is significantly associated with PNA
Soubasi et al. (2009)	96 preterm infants (GA: 30.18 ± 2 weeks)	aEEG recordings	• Preterm infants exhibit definite sleep-wake cycles with advanced GA
			• The evolution of sleep-wake cycling is correlated with positive significant interaction of PMA and GA
Foreman et al. (2008)	97 preterm infants (GA: 32.72 ± 2.28 weeks)	Video recordings	• With advancing PMA for preterm infants: AS↓, QS↑, drowsy↑, awake↑, defined states↑, diffuse states↓
			• Male *vs.* female preterm infants: AS↓, drowsy↑, awake↑, defined states↓, diffuse states↑
Sisman et al. (2005)	31 preterm infants (GA: 25–32 weeks)	aEEG recordings	• The frequency of mature sleep-wake cycling in preterm infants increased with PMA independent of GA
Scher et al. (2005)	33 preterm infants (GA: 23–29 weeks)	EEG-sleep recordings	• Most preterm infants exhibit about 37–100 min period ultradian sleep state rhythms at 25–30 weeks PMA
Hoppenbrouwers et al. (2005)	195 preterm infants (GA: 30.5 ± 3.3 weeks) *vs.* 88 term infants (GA: 39.4 ± 1.0 weeks)	Polysomnography	• Preterm *vs.* term infants: AS↑, QS↓, SE↓
			• With advancing PMA for preterm infants: AS↓, QS↑, SE↑
			• Preterm infants’ sleep-wake architecture is associated with ventilatory support, gestational age, and maternal smoking, but without sex or steroid administration
Holditch-Davis et al. (2004)	134 preterm infants (GA: 28.8 ± 2.6 weeks)	Direct behavioral observations	• With advancing PMA for preterm infants: AS↓, QS↑, quiet and active waking states↑, large body movements↓
			• Sleep-wake transitions in preterm infants increased until 40 weeks PMA and changed to decrease after 43 weeks PMA
Mirmiran et al. (2003b)	40 preterm infants (GA: 30.2 ± 1.5 weeks)	Video recordings	• With advancing PMA for preterm infants: AS↓, QS↑
Korte et al. (2001)	10 preterm infants (GA: 34–36 weeks) *vs.* 10 term infants (GA: 37–42 weeks)	Actigraphy; Standardized diaries	• Preterm *vs.* term infants: ultradian activity-rest rhythms↑, circadian activity-rest rhythms↓, no difference in TST
			• With advancing PMA for preterm infants: nighttime sleep↑, daytime sleep↓
Bach et al. (2000)	38 preterm infants (GA: 34 ± 2 weeks)	EEG; Eye movement recordings	• Cool exposure leads to: TST↓, longest sleep period↓, wakefulness↑, AS↑, QS↓
			• Male *vs.* female preterm infants: TST↓, longest sleep period↓, wakefulness↑, AS↑, QS↓
Antonini et al. (2000)	9 preterm infants (GA: 31.3–34.6 weeks)	Sleep diagrams by the mother	• With advancing PNA for preterm infants: daytime sleep↓, nighttime sleep↑, TST unchanged, nighttime sleep > daytime sleep after 8 weeks PNA
Shimada et al. (1999)	44 preterm infants (GA: 31.0 ± 3.4 weeks) *vs.* 40 term infants (GA: 39.6 ± 1.3 weeks)	Sleep diagrams by the mother; Parental sleep questionnaires; Video recordings	• 75% of these preterm infants have an ultradian or irregular sleep-wake rhythms unrelated to feeding for 3–4 weeks after discharge from the hospital
			• Circadian sleep-wake rhythms in preterm infants were entrained at the mean age of approximately 45 weeks PMA, similar as term infants
Ingersoll and Thoman, (1999)	95 preterm infants (GA: 28.5 ± 2.2 weeks)	Video recordings	• With advancing PMA for preterm infants: QS↑, AS↓, wakefulness↓, bout lengths of QS↑, bout lengths of AS and wakefulness do not change
Sahni et al. (1995)	35 preterm infants (GA: 31.0 ± 2.0 weeks)	Direct behavioral observations; EEG	• Preterm infants spend about 75% of their sleep time in AS and 19% in QS between 30 and 39 weeks PMA
			• With advancing PMA for preterm infants: AS↓, QS↑
Glotzbach et al. (1995)	17 preterm infants (GA: 31.1 ± 1.2 weeks)	Actigraphy	• Preterm infants exhibit feeding-related ultradian sleep-wake rhythms at about 35 weeks PMA
Borghese et al. (1995)	49 preterm infants (GA: 28.6 ± 2.6 weeks)	Motility monitoring system	• Most preterm infants exhibit both ultradian and diurnal sleep-wake rhythms at 36 weeks PMA
			• From 36 weeks to 6 months PMA: QS↑, wakefulness↑, AS↓, frequency and degree of within-sleep cyclicity↑
Ardura et al. (1995)	60 preterm infants (GA: 33.4 ± 2.4 weeks) *vs.* 63 term infants (GA: 39.5 ± 1.3 weeks)	Direct behavioral observations	• Preterm *vs.* term infants: TST↑, daytime sleep↑, nighttime sleep↑
			• With advancing PMA for preterm infants: TST↓, daytime sleep↓, nighttime sleep does not change
Hayes et al. (1993)	13 preterm infants (GA: 26–36 weeks)	Actigraphy	• Preterm infants exhibit 80 min and 30 min periods ultradian activity state rhythms
			• With advancing PMA for preterm infants: ultradian periodicities↓, activity bout durations↑
Curzi-Dascalova et al. (1993)	24 preterm infants (GA: 26.3–34.1 weeks)	Polysomnography	• Preterm infants spend most of their sleep time in AS rather QS after 27 weeks PMA
			• With advancing PMA for preterm infants: AS↓, QS↓, IS↑
Mirmiran and Kok, (1991)	12 preterm infants (GA: 25–32 weeks)	Actigraphy	• Only one of these preterm infants exhibit the 24-h period circadian rest-activity rhythms at 29 weeks PMA
McMillen et al. (1991)	19 preterm infants (GA: 27–35 weeks) *vs.* 22 term infants (GA: 38–42 weeks)	Sleep-wake activity diaries	• PNA at the circadian sleep-wake rhythms entrained are inversely correlated with GA for preterm infants, with 50% of preterm infants begin to exhibit circadian rhythms at 47 weeks PMA
			• Preterm *vs.* term infants: earlier PMA at circadian rhythms entrained
Mirmiran et al. (1990)	11 preterm infants (GA: 26–32 weeks)	Actigraphy	• Preterm infants exhibit ultradian rest-activity rhythms rather than circadian rhythms at 28–35 weeks PMA
Curzi-Dascalova et al. (1988)	18 preterm infants (GA: 34.2 ± 0.5 weeks) *vs.* 20 term infants (GA: 38.8 ± 0.2 weeks)	Polysomnography	• With advancing PMA for preterm infants: mean sleep cycle duration↑, AS↑, QS↑, IS↓
Anders and Keener, (1985)	24 preterm infants (GA: 27–35 weeks) *vs.* 40 term infants (GA: >37 weeks)	Video recordings	• Preterm *vs.* term infants: TST↑, LSP↑, AS↑, QS↓
			• With advancing PMA for preterm infants: TST↑, LSP↑, AS↓, QS↑, wakefulness↑

Abbreviations: aEEG, amplitude-integrated electroencephalography; AS, active sleep; EEG, electroencephalography; GA, gestational age; IS, indeterminate sleep; LSP, longest sustained sleep period; PMA, postmenstrual age; PNA, postnatal age; QS, quiet sleep; SE, sleep efficiency; TST, total sleep time.

**TABLE 2:** Studies about the cardiorespiratory rhythms in preterm infants.

Studies	Subjects	Methods of evaluation	Main findings
Koch et al. (2021)	65 preterm infants (GA: 30.8 ± 2.1 weeks)	Surface EMG	• The base HR are negatively correlated with GA during the first 5 days of life
			• Average oscillating period length of HR rhythms: 159 min
			• Average amplitude of HR rhythms: 5.9 bpm
Hasenstab-Kenney et al. (2020)	40 preterm infants (GA: 27.0 ± 3.1 weeks)	Respiratory inductance plethysmography; Nasal thermistor; ECG	• Pharyngeal irritation leads to: HR↓, duration of cardiac rhythms responses↑, respiratory rhythms changes↑
Hasenstab et al. (2019)	48 preterm infants (GA: 27.7 ± 0.5 weeks)	Respiratory inductance plethysmography; Nasal thermistor; ECG	• Pharyngeal stimulation leads to HR decreased in 32% preterm infants and remained stable in 61%
			• HR decrease is related to extreme prematurity and resulted in increased respiratory rhythms disturbance
Bauer et al. (2009)	22 preterm infants (GA: 30.3 ± 1.7 weeks)	Indirect calorimetry	• Oxygen consumptions are significantly associated with the HR
			• Circadian rhythms of oxygen consumptions with two peaks in the afternoon and early morning are detected in most preterm infants early after birth
Gewolb and Vice, (2006)	20 preterm infants (GA: 29.4 ± 2.1 weeks) *vs.* 16 term infants (GA: 39.2 ± 1.1 weeks)	Pharyngeal pressure transducer; Thoracoabdominal strain gauge	• With advancing PMA for preterm infants: percentage of apneic swallows↓, variation of breath interval↓, integration of swallow and respiratory rhythms↑
			• Stabilization of suck and suck-swallow rhythms occurs at about 36 weeks PMA, and coordination of respiration and swallow rhythms occurs later
Begum et al. (2006)	124 preterm infants (GA: 23–36 weeks) *vs.* 63 term infants (GA: 37–42 weeks)	ECG; Pulse oximetry	• Circadian cycles are observed among 23.8% neonates in HR, 20% in PR, 27.8% in RR, and 16% in SpO_2_ in first 3 days of life
			• Percentages of circadian PR cycles are negatively correlated with GA, but amplitudes are positively correlated with GA and PMA
Gewolb et al. (2001)	20 preterm infants (GA: 29.4 ± 2.1 weeks)	Pharyngeal pressure transducer; Nasal thermistor; Cardiac monitor	• Swallow rhythms are stable after 32 weeks PMA, percentage of swallows in runs increased with increasing PMA
			• Stability of suck rhythms and sucks in runs are positively correlated with PMA
Dimitriou et al. (1999)	22 preterm infants (GA: 23–28 weeks)	Indwelling arterial cannula transducer	• Significant circadian and ultradian rhythms of BP are shown on day 2 but not day 7 after birth
Glotzbach et al. (1995)	17 preterm infants (GA: 31.1 ± 1.2 weeks)	ECG	• Preterm infants exhibit feeding-related ultradian HR rhythms at about 35 weeks PMA
D'Souza et al. (1992)	9 preterm infants (GA: 26–29 weeks)	Skin electrodes monitor	• Three preterm infants exhibit circadian HR rhythms at 33–42 weeks PMA
Tenreiro et al. (1991)	20 preterm infants (GA: 24–29 weeks)	Surface electrode monitor	• Circadian and ultradian HR rhythms are appeared and disappeared erratically for the period of 6–17 weeks after birth
			• Circadian and ultradian rhythmicity of HR increases with regular light-dark and feeding patterns
Mirmiran and Kok, (1991)	12 preterm infants (GA: 25–32 weeks)	Neonatal intensive care monitor	• Five of these preterm infants exhibit the 24-h period circadian HR rhythms at 29–33 weeks PMA
Updike et al. (1985)	6 preterm infants (GA: 34–37 weeks)	Noninvasive electrodes monitor	• Three preterm infants exhibit circadian respiratory pause frequency rhythms with peak occurring between 23:00 to 05:00 during 10–20 days after birth
			• Two preterm infants exhibit circadian transcutaneous oxygen level rhythms with trough occurring between 00:30 to 04:30 during 10–20 days after birth

Abbreviations: BP, blood pressure; bpm, beats per minute; ECG, electrocardiography; EMG, electromyography; GA, gestational age; HR, heart rate; PMA, postmenstrual age; PR, pulse rate; RR, respiratory rate; SpO_2_, pulse oximeter oxygen saturation.

**TABLE 3:** Studies about the body temperature rhythms in preterm infants.

Studies	Subjects	Methods of evaluation	Main findings
Koch et al. (2021)	65 preterm infants (GA: 30.8 ± 2.1 weeks)	Zero heat flux method *via* the skin electrode	• Average oscillating period length of ultradian BT rhythms within the first 5 days of life: 290 min
			• Average amplitude of BT rhythms: 0.147°C
Bueno and Menna-Barreto, (2016)	19 preterm infants (GA: 28–36 weeks)	Wrist skin thermistor record	• Dominant circadian WT rhythms are present in preterm infants since the first 2 weeks of life
Mirmiran et al. (2003b)	40 preterm infants (GA: 30.2 ± 1.5 weeks)	Rectal digital ambulatory record	• Preterm infants mainly exhibit 2–4 h period ultradian BT rhythms at 36 weeks PMA
			• Preterm infants exhibit 12 and 24 h period circadian BT rhythms at 1–3 months after birth
			• The amplitude of BT rhythms is correlated with PMA and light-dark patterns
Thomas, (2001)	26 preterm infants (GA: 30.9 ± 2.1 weeks)	Skin transducer monitor	• 21 preterm infants exhibit circadian BT rhythms at mean of 33 weeks PMA
			• The amplitude of BT rhythms is correlated with PMA for not sick infants, but not for sick infants
Thomas and Burr, (2002)	34 preterm infants (GA: 26–33 weeks)	Abdominal skin thermistor record	• Preterm infants have circadian ST rhythms at 44–46 weeks PMA
			• The acrophase of circadian ST rhythms is related to parental co-sleeping and hospital stay length
Glotzbach et al. (1995)	17 preterm infants (GA: 31.1 ± 1.2 weeks)	Rectal and abdominal skin thermistor record	• Preterm infants exhibit feeding-related ultradian RT and ST rhythms at about 35 weeks PMA
			• Amplitudes of RT rhythms of preterm infants at 35–37 weeks PMA are much higher compared with 32–34 weeks PMA
D'Souza et al. (1992)	9 preterm infants (GA: 26–29 weeks)	Skin electrodes monitor	• Four of these preterm infants exhibit light-related circadian ST rhythms at 34–42 weeks PMA
Tenreiro et al. (1991)	20 preterm infants (GA: 24–29 weeks)	Surface electrode monitor	• Circadian and ultradian ST rhythms are appeared and disappeared erratically during 6–17 weeks after birth
			• Circadian and ultradian rhythmicity of ST increases with regular light-dark and feeding patterns
Mirmiran and Kok, (1991)	12 preterm infants (GA: 25–32 weeks)	Skin transducer monitor	• Seven of these preterm infants exhibit circadian BT rhythms with different periods and out of time synchronization at 29–34 weeks PMA
Mirmiran et al. (1990)	11 preterm infants (GA: 26–32 weeks)	Rectal sensor monitor	• Five preterm infants exhibit circadian RT rhythms with high values at night and low values during the day at 28–34 weeks PMA
Updike et al. (1985)	6 preterm infants (GA: 34–37 weeks)	Skin thermistor record	• Five preterm infants exhibit circadian ST rhythms with trough occurring between 23:00 to 04:30 during 10–20 days after birth

Abbreviations: BT, body temperature; GA, gestational age; PMA, postmenstrual age; RT, rectal temperature; ST, skin temperature; WT, wrist temperature.

**TABLE 4:** Studies about the hormonal rhythms in preterm infants.

Studies	Subjects	Methods of evaluation	Main findings
Biran et al. (2019)	209 preterm and term infants (GA: 24.0–41.9 weeks)	Plasma melatonin and urine 6-sulfatoxymelatonin levels by RIA	• No obvious rhythms of plasma melatonin and urine 6-sulfatoxymelatonin excretion were found in these neonates during first 55 days of life
Ivars et al. (2017)	51 preterm infants (GA: 23.3–31.9 weeks) *vs.* 130 term infants (GA: 37–42 weeks)	Salivary cortisol levels by RIA	• Salivary cortisol circadian rhythms in preterm infants are established by 1 month CA and persisted throughout the first year
			• The establishment of salivary cortisol circadian rhythms is correlated with GA and delayed by topical corticosteroid medication
Dorn et al. (2014)	60 preterm infants (GA: 33.0 ± 10.8 weeks)	Salivary cortisol levels by ELISA	• No circadian or ultradian rhythms of salivary cortisol are found in preterm infants during the first 3 weeks of life except one at 34.3 weeks PMA
			• Salivary cortisol levels in day 1 are higher than day 7 and 14 after birth, nighttime cortisol levels are higher than daytime
Kidd et al. (2005)	11 preterm infants (GA: 26–29 weeks)	Salivary cortisol levels by RIA	• No circadian salivary cortisol rhythms are found during the first 4 weeks of life
			• Five infants exhibit unsustainable adult-type rhythms after 39 weeks PMA
			• Salivary cortisol levels are negatively correlated with PNA
Antonini et al. (2000)	9 preterm infants (GA: 31.3–34.6 weeks)	Salivary cortisol levels by RIA	• Salivary cortisol circadian rhythms in preterm infants are emerged and persisted at approximately 8–12 weeks after birth
Jett et al. (1997)	14 preterm infants (GA: 25.6 ± 1.3 weeks)	Plasma cortisol levels by RIA	• No circadian rhythm of plasma cortisol is found in preterm infants during the first 4 days of life
Mantagos et al. (1996)	23 preterm infants (GA: 33–36 weeks)	Plasma melatonin levels by RIA	• No circadian rhythm of plasma melatonin is found in preterm infants under cyclic or constant light conditions during the first 4 days of life
Commentz et al. (1996)	64 preterm and term male infants (GA: 26–42 weeks)	Urine melatonin and 6-hydroxymelatonin sulfate levels by RIA	• No circadian rhythm of urine melatonin and 6-hydroxymelatonin sulfate excretion are found in these infants during the first 7 days of life
			• Urine melatonin and 6-hydroxymelatonin sulfate excretion in these infants are negatively correlated with GA
Economou et al. (1993)	60 preterm and term infants (GA: 33.5 ± 1.5 weeks)	Serum cortisol levels by IFA	• A free running serum cortisol rhythm is found in healthy preterm and term infants during the first 4 weeks of life
			• Sick preterm and term infants exhibit higher serum cortisol levels at 20:00, while healthy infants exhibit lower levels at 20:00
Kennaway et al. (1992)	14 preterm infants (GA: 29–35 weeks) *vs.* 17 term infants (GA > 37 weeks)	Urine 6-sulfatoxymelatonin levels by RIA	• Appearance of rhythmic urine 6-sulfatoxymelaton in preterm infants are delayed by 9 weeks than term infants and 2–3 weeks after correcting for GA
			• Urine 6-sulfatoxymelaton excretion in preterm infants is gradually increased during the first 52 weeks after birth but lower than term infants

Abbreviations: CA, corrected age; ELISA, enzyme linked immune sorbent assay; GA, gestational age; IFA, immunofluorescence assay; PMA, postmenstrual age; PNA, postnatal age; RIA, radioimmunoassay.

### 5.1 Sleep-wake rhythms

It is well established that the sleep is essential for normal brain development and health throughout the whole life (Peirano et al., 2003; Gogou et al., 2019). Premature newborns spend more than 70% of their first several weeks sleeping after birth (Ardura et al., 1995; Wong et al., 2022), thereby maintaining the proper sleep homeostasis is even more important for their neurological development and functional maturation (Bennet et al., 2018; Uchitel et al., 2021). The direct behavioral observations, parental sleep questionnaires, video recordings, polysomnography, actigraphy, and electroencephalography (EEG) (Table 1) have been developed to investigate the sleep-wake states of neonates (Mirmiran et al., 2003a; Collins et al., 2015; Gogou et al., 2019).

Based on the behavioral, cardiopulmonary, and EEG patterns (Darnall et al., 2006; Dereymaeker et al., 2017), the sleep states of preterm infants are generally classified as: active sleep (AS), the precursor of adult rapid eye movement (REM) sleep; quiet sleep (QS), the precursor of adult non-REM sleep; and indeterminate sleep (IS), the transition between AS and QS patterns (Mirmiran et al., 2003a; Liao et al., 2018). More specifically, the AS could promote the synapse formation, neuronal differentiation and migration, and the development of brain functional connectivity networks (Kurth et al., 2017; Gogou et al., 2019), whilst the QS promote the myelination, replenishment of energy reserves, and cognitive development in premature infants (Liao et al., 2018; Gogou et al., 2019).

As summarized in Table 1, Curzi-Dascalova et al. (1993), found the AS and QS states can be discerned in preterm infants as early as 27 weeks of GA. The results varied due to the different GA of the enrolled cases, but most studies revealed that preterm infants experienced more total sleep time and AS, while less QS than term ones (Anders and Keener, 1985; Ardura et al., 1995; Sahni et al., 1995; Hoppenbrouwers et al., 2005; Guyer et al., 2015; Georgoulas et al., 2021), which might reflect the accelerated neurological maturation of preterm infants (Mirmiran et al., 2003a; Bennet et al., 2018). Besides, preterm infants had fewer total arousals and, more specifically, fewer arousals in the AS (Guyon et al., 2022), which seemed to cause a higher risk of sudden infant death syndrome (Mirmiran et al., 2003a; Bennet et al., 2018).

With developmental maturity, preterm infants have more sleep during nighttime but less during daytime (Antonini et al., 2000; Korte et al., 2001; Guyer et al., 2015; Lan et al., 2019; Guyon et al., 2022). Meanwhile, as the PMA increased, the AS proportion comes out of a decreasing trend, but it is not true for the QS, IS, wakefulness, and activity, which all experience an increasing trend (Anders and Keener, 1985; Curzi-Dascalova et al., 1988; Curzi-Dascalova et al., 1993; Borghese et al., 1995; Sahni et al., 1995; Ingersoll and Thoman, 1999; Mirmiran et al., 2003b; Holditch-Davis et al., 2004; Hoppenbrouwers et al., 2005; Foreman et al., 2008; Dorn et al., 2014; Guyer et al., 2015; Lan et al., 2019; Cailleau et al., 2020; Park et al., 2020; Georgoulas et al., 2021; Guyon et al., 2022). In addition, other factors like sex, illness severity, body weight, ventilatory support, maternal smoking, and ambient temperature also affect the sleep-wake patterns (Bach et al., 2000; Hoppenbrouwers et al., 2005; Foreman et al., 2008; Lan et al., 2019).

It is well understood that the sleep homeostasis in humans are regulated by two independent but synergistic processes (Borbély, 1982; Deboer, 2018): a Clock-dependent circadian process (Process C), controlled by the SCN circadian oscillator, determines the alternation of different sleep propensity (Cremer et al., 2016); and a Sleep-dependent homeostatic process (Process S) that is determined by the prior sleep pressure, which comes from the adenosine buildup in the basal forebrain during wakefulness (Deboer, 2018; Wong et al., 2022). However, due to the immature development of the central nervous system, especially the SCN, Process C and Process S are not stably present in preterm infants or even in term ones (Salzarulo and Fagioli, 1992; Schwichtenberg et al., 2016). As a result, preterm infants experience many sleep and wake episodes within the 24-h period, and those ultradian sleep-wake rhythms persist for several months until the Process C and Process S are gradually developed (Mirmiran et al., 2003a; Cremer et al., 2016).

As shown in Table 1, preterm infants exhibit ultradian or irregular sleep-wake rhythms with different periods in the early postnatal life (Mirmiran et al., 1990; Hayes et al., 1993; Borghese et al., 1995; Shimada et al., 1999; Scher et al., 2005; Dorn et al., 2014; Koch et al., 2021), which might be explained by the environmental factors, such as feeding patterns (Glotzbach et al., 1995; Thomas, 2000; Bueno and Menna-Barreto, 2016) and respiratory states (Palmu et al., 2013). As for when the sleep-wake rhythms begin to occur and entrain, Scher et al. (2005), observed the ultradian sleep-wake rhythms as early as 25 weeks of PMA. Mirmiran and Kok, (1991) found the circadian sleep-wake rhythms began to appear after 29 weeks of PMA. However, McMillen et al. (1991), found that the entrainment of circadian sleep-wake rhythms did not occur in 50% of the preterm infants at 47 weeks of PMA, and all cases did not begin to develop the circadian rhythms until approximately 54 weeks of PMA.

Besides, several studies also demonstrated that a definite sleep-wake cycling existed in preterm infants with the advanced GA and became more prominent as the PMA increased (Sisman et al., 2005; Soubasi et al., 2009; Lee et al., 2010). Therefore, it could be concluded that with the continuous development of the brain and neural functions, circadian sleep-wake rhythms in preterm infants are consolidated and eventually developed to a 24-h pattern, just as those in adults (Mirmiran et al., 2003a; Bennet et al., 2018).

### 5.2 Cardiorespiratory rhythms

Many physiological biomarkers of the cardiopulmonary system in adults, such as the heart rate, blood pressure, and respiratory rate, exhibit distinct circadian rhythms (Elstad et al., 2018). A complex network that composed of the brainstem respiratory center, autonomic nervous system, and a variety of central and peripheral chemoreceptors and mechanoreceptors is responsible for regulating the rhythmic oscillations of the cardiorespiratory system (Darnall et al., 2006; Longin et al., 2006). Due to the immaturity of this network (Hunt, 2006), cardiorespiratory events like apnea, periodic breathing, and bradycardia are common in premature infants (Hodgman et al., 1990; Darnall et al., 2006), which leads to the erratic cardiopulmonary rhythms with marked individual differences (Begum et al., 2006). Clinically, the incidence and duration of cardiorespiratory events are associated with the GA and PMA (Hellmeyer et al., 2012; Fairchild et al., 2016; Patel et al., 2016).

As shown in Table 2, some, but not all, preterm infants experienced circadian or ultradian rhythms for the heart rate, pulse rate, respiratory rate, blood pressure, and oxygen consumption at the first few weeks after birth (Begum et al., 2006; Mirmiran and Kok, 1991; Bauer et al., 2009; D'Souza et al., 1992; Updike et al., 1985). Interestingly, unlike the ultradian sleep-wake rhythms gradually grew into circadian rhythms after birth, these cardiopulmonary rhythms in premature infants appeared and disappeared erratically (Tenreiro et al., 1991; Dimitriou et al., 1999), *e.g.*, presence on day 2 but absence on day 7 after birth for the heart rate rhythms, which might be caused by the residual of maternal effects (Dimitriou et al., 1999). Tenreiro et al. (1991) also proposed that the circadian components of these cardiopulmonary rhythms gradually and erratically came into phases with one another, while the regular light-dark and feeding patterns seemed to promote the presence of the dominant circadian rhythms, which developed as the increased coupling between the component oscillators.

In addition, the well-developed laryngeal reflexes and coordination of pharyngoesophageal-cardiorespiratory (PECR) responses are essential for the development and maintenance of cardiorespiratory rhythms (Gewolb and Vice, 2006; Hasenstab-Kenney et al., 2020). As shown in Table 2, pharyngeal stimulations cause a decrease of heart rate in premature infants with uncoordinated suck-swallow-respiration rhythms due to the immature laryngeal reflexes and PECR responses, which would aggravate the disturbance of cardiac and respiratory rhythms (Hasenstab et al., 2019; Hasenstab-Kenney et al., 2020). (Gewolb et al., 2001; Gewolb and Vice, 2006) found that the development and establishment of suck-swallow rhythms were associated with their PMA. The swallow rhythms appeared at 32 weeks of PMA first (Gewolb et al., 2001), followed by the stabilization of suck and suck-swallow rhythms between 36 and 40 weeks of PMA (Gewolb and Vice, 2006), then the suck-swallow-respiration rhythms began to coordinate and to integrate as the adaptation of feeding patterns and the maturation of neurodevelopment (Darnall et al., 2006).

### 5.3 Body temperature rhythms

The human body temperature is precisely regulated by a network that consists of the skin thermal sensors, hypothalamic thermoregulatory center, autonomic nervous system, and several thermoregulation effector systems including brown adipose tissue, peripheral vasomotricity, and sweat glands (Bach et al., 1996; Jost et al., 2017). Due to the immaturity of the regulatory network, especially the dysfunction of the autonomic nervous system, their body temperature during the first few days of life is susceptible to the rapidly changed external environment temperature (Jost et al., 2017). Therefore, premature infants are typically nursed in the incubators to treat the autonomic dysregulation of body temperature (Thomas, 2001). Interestingly, Bueno and Menna-Barreto (2016), found a positive correlation between the wrist temperature and environment temperature inside the incubator, but no significant association between the period or potency for them. Similarly, Thomas (2001) demonstrated that the circadian of incubator temperature did not appear to be the primary determinant of the body temperature rhythms.

As summarized in Table 3, due to the heterogeneity of the body temperature monitoring, the GA of preterm infants, and sample size, the body temperature rhythms have not yet been consistently described. Several studies observed the ultradian body temperature rhythms within the first few days of life (Glotzbach et al., 1995; Mirmiran et al., 2003b; Koch et al., 2021), and the circadian rhythms by approximately 1–3 months of PNA (Mirmiran et al., 2003b; Bueno and Menna-Barreto, 2016). Interestingly, Thomas and Burr (2002), found that the acrophase of circadian abdominal skin temperature rhythms was related to the parental co-sleeping and length of hospital stay for preterm infants at 44–46 weeks of PMA. However, some studies demonstrated that the body temperature rhythms were only found in some, but not all preterm infants (Mirmiran et al., 1990; Mirmiran and Kok, 1991; D'Souza et al., 1992; Updike et al., 1985; Thomas, 2001). For example, Tenreiro et al. (1991) found that the ultradian and circadian rhythms of skin temperature appeared and disappeared erratically during 6–17 weeks of PNA, which was similar to the cardiopulmonary rhythms.

### 5.4 Hormonal rhythms

As summarized in Table 4, due to the difficulties in sample collection and analysis, studies on hormonal rhythms in preterm infants are still very limited until now, and nearly all focused on the cortisol and melatonin rhythms. With regard to the cortisol, due to the immature of HPA axis (Bolt et al., 2002), no significant circadian or ultradian rhythms were observed during the early postnatal periods (Economou et al., 1993; Jett et al., 1997; Kidd et al., 2005; Dorn et al., 2014). Nevertheless, studies have found that healthy preterm infants had higher nighttime cortisol levels than daytime at birth, and that cortisol levels tended to decrease gradually after birth (Kidd et al., 2005; Dorn et al., 2014). Impressively, premature infants with perinatal stress like respiratory distress experienced higher cortisol levels at nighttime after birth compared with those healthy preterm and term neonates (Economou et al., 1993; Gunes et al., 2006).

It remains unclear when premature infants develop the circadian cortisol rhythms. Antonini et al. (2000) found the salivary cortisol circadian rhythms emerged and persisted at approximately 8–12 weeks of PNA, which was in line with term infants. However, Ivars et al. (2017) found that the cortisol rhythms were established by 1 month of corrected age, persisted throughout the first year of life, but delayed by topical corticosteroid medication. In addition, Ivars et al. (2017) also suggested that the establishment of cortisol rhythms was related to the GA rather than PNA, because the maturation of adrenal cortex was depend on the GA of preterm infants (Bolt et al., 2002).

Circadian melatonin rhythms could not be detected in preterm infants under different ambient illumination conditions during the early postnatal life (Commentz et al., 1996; Mantagos et al., 1996; Biran et al., 2019). Several studies demonstrated that the blood melatonin and urine 6-sulfatoxymelatonin levels were positively correlated with the GA (Biran et al., 2019) and birth weight of preterm infants (Muñoz-Hoyos et al., 2007), but the serum melatonin levels and urine 6-sulfatoxymelaton excretion increased during the first 7 days and even 52 weeks of PNA (Kennaway et al., 1992; Commentz et al., 1996; Muñoz-Hoyos et al., 2007), which might be attributed to the gradual maturation of the pineal gland where the melatonin is mainly synthesized (Commentz et al., 1997).

However, Commentz et al. (1996) found the urine melatonin and 6-hydroxymelatonin sulfate excretion in male preterm infants during 2–7 days of PNA were negatively associated with the GA, indicating that the melatonin levels might be related to the sex. As for the establishment of circadian melatonin rhythms, Kennaway et al. (1992) observed the appearance of urine 6-sulfatoxymelaton circadian rhythms was approximately at 18–21 weeks of PNA, which was delayed by 9 weeks than those term infants and 2–3 weeks after correcting for GA.

## 6 The effects and mechanisms of caffeine on circadian rhythms

The potential association between caffeine consumption and circadian rhythms has attracted extensive attention in the past decades (Landolt, 2015). However, the underlying mechanisms remain largely elusive. Various research attempts in the non-human field also reinforce this impression (Spaeth et al., 2014). In this section, we briefly introduce the up-to-date progress that achieved in human and non-human mammals, while the effects on premature infants will be delineated in the next section.

### 6.1 The effects of caffeine on circadian rhythms

In humans, several clinical observational studies with small sample size have witnessed the alterations of circadian sleep-wake (Landolt et al., 1995a; Landolt et al., 1995b; McHill et al., 2014; Weibel et al., 2021), body temperature (Wright et al., 1997; Wright et al., 2000; McHill et al., 2014), blood pressure (Green and Suls, 1996; Guessous et al., 2014), heart rates (Green and Suls, 1996; Kohler et al., 2006; Crooks et al., 2019), melatonin (Wright et al., 1997; Wright et al., 2000; Burke et al., 2015), and cortisol rhythms (Lovallo et al., 2005; Rieth et al., 2016) in adults who consumed caffeine by comparison with placebo controls.

In rodents, caffeine disrupted the mesors, amplitudes, and acrophases of the circadian heart rate, temperature, motor activity, and sleep-wake rhythms (Pelissier et al., 1999; Pelissier-Alicot et al., 2002; Vivanco et al., 2013; Panagiotou et al., 2019). Caffeine also potentiated the light-induced phase shift, which responded to the rest-activity circadian rhythms, indicating that caffeine enhanced the clock sensitivity to light (Antle et al., 2001; Vivanco et al., 2013; van Diepen et al., 2014; Jha et al., 2017; Ruby et al., 2018). In addition, caffeine lengthened the period and amplitude of circadian clocks in mammalian cells *in vitro* and in mice *ex vivo* and *in vivo* (Oike et al., 2011; Narishige et al., 2014; Burke et al., 2015). At the cellular level, caffeine also altered the expression of circadian clock genes, such as *Clock*, *Bmal1*, and *Per1* in the liver and jejunum of mice under *ad libitum* feeding conditions (Sherman et al., 2011).

### 6.2 The mechanisms of caffeine on circadian rhythms

**FIGURE 3:**
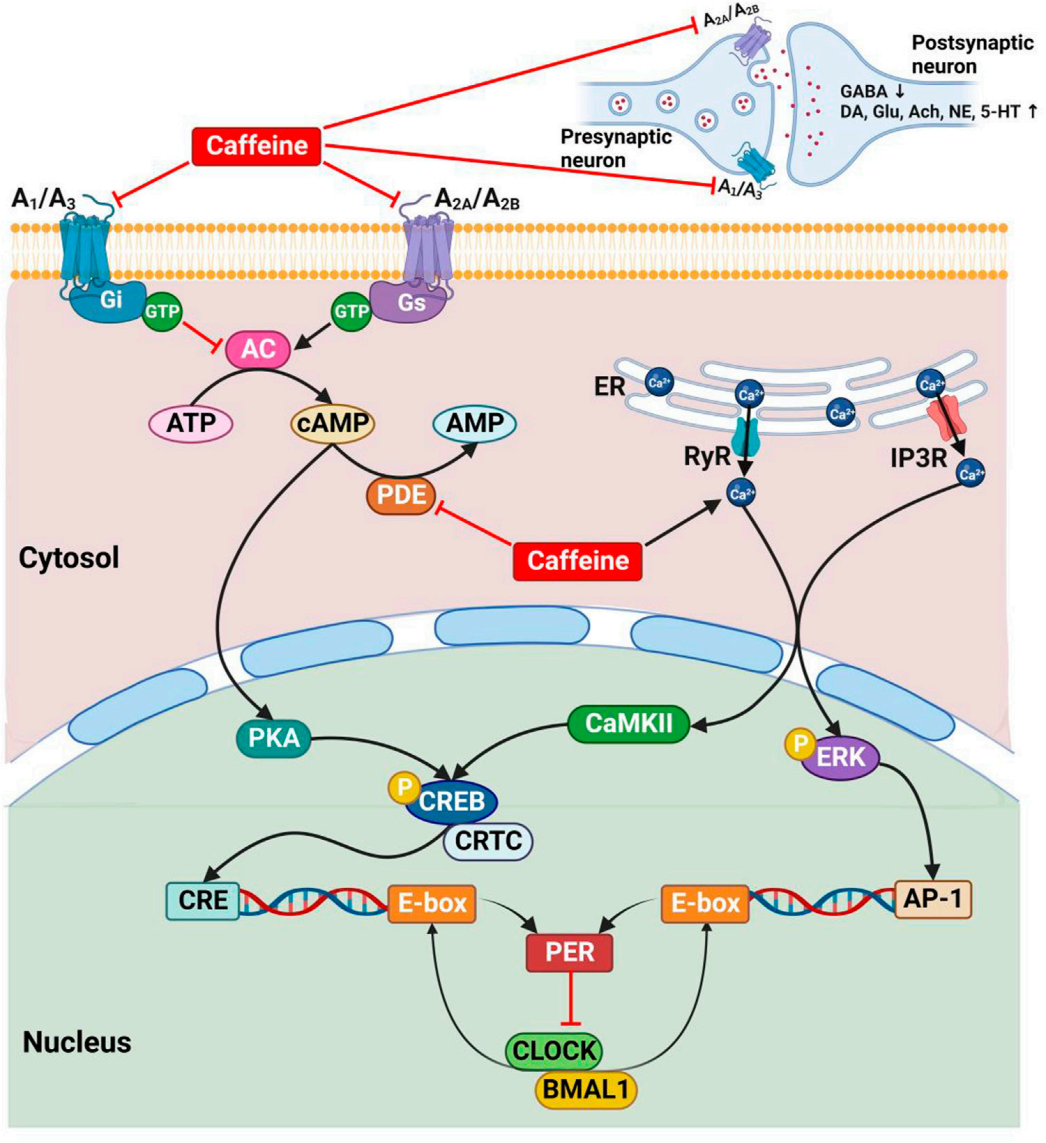
The mechanisms of caffeine on circadian rhythms. A_1_, A_3_, A_2A_, and A_2B_, adenosine receptors; AC, adenylate cyclase; Ach, acetylcholine; AMP, adenosine monophosphate; ATP, adenosine triphosphate; AP-1, activator protein 1; BMAL1, brain and muscle ARNT-like 1; CaMKⅡ, Ca^2+^/calmodulin-dependent protein kinase Ⅱ; cAMP, cyclic adenosine monophosphate; CLOCK, circadian locomotor output cycles kaput; CRE, cAMP response element; CREB, cAMP responsive element binding protein; CRTC, CREB regulated transcription coactivator; DA, dopamine; ER, endoplasmic reticulum; ERK, extracellular regulated protein kinases; GABA, γ-aminobutyric acid; Gi, inhibitory adenylate cyclase G protein; Glu, glutamate; Gs, stimulating adenylate cyclase G protein; GTP, guanosine triphosphate; IP3R, inositol triphosphate receptor; NE, norepinephrine; P, phosphorylation; PDE, phosphodiesterase; PER, period; PKA, protein kinase A; RyR, ryanodine receptor; 5-HT, serotonin.

Caffeine influences the circadian rhythms by modulating the endogenous cAMP/Ca^2+^ signaling pathway, the core components of the mammalian circadian pacemaker (Harvey et al., 2020; O'Neill et al., 2008), through a variety of complex mechanisms (Aguilar-Roblero et al., 2007; Narishige et al., 2014; Burke et al., 2015; Landolt, 2015; Jagannath et al., 2021) (Figure 3). Basically, caffeine antagonizes all types of adenosine receptors (A_1_, A_2A_, A_2B_, and A_3_ receptors) and mainly functions by non-specifically antagonizing the A_1_ and A_2A_ receptors (Nehlig et al., 1992; Cappelletti et al., 2015; Rodak et al., 2021; Yang et al., 2021). The blockade of adenosine receptors indirectly regulates the production of cAMP by inhibition (A_1_ and A_3_ receptors) or stimulation (A_2A_ and A_2B_ receptors) of adenylate cyclase (Nehlig et al., 1992; Kumar and Lipshultz, 2019; Yang et al., 2021). Caffeine also prevents the degradation and increases the intracellular cAMP levels by non-selectively inhibiting phosphodiesterase (Nehlig et al., 1992; Cappelletti et al., 2015; Kumar and Lipshultz, 2019; Yang et al., 2021). In addition, caffeine mobilizes intracellular Ca^2+^ transmission from the endoplasmic reticulum through activating the ryanodine receptor channels (Aguilar-Roblero et al., 2007; Kumar and Lipshultz, 2019) and the inositol triphosphate receptors (Yang et al., 2021).

The increased cytosolic cAMP/Ca^2+^ activates the protein kinase A (PKA) and Ca^2+^/calmodulin-dependent protein kinase Ⅱ (CaMKⅡ), thereby leading to the phospho-dependent activation of cAMP response element binding protein (CREB), which in concert with its coactivators to activate the cAMP response element (CRE) (Narishige et al., 2014; Harvey et al., 2020; Reichert et al., 2022). Besides, the increased intracellular Ca^2+^ levels also result in the phosphorylation of extracellular regulated protein kinases (ERK), which drives to form the activator protein 1 (AP-1) transcription factor (Jagannath et al., 2021). Then, interestingly, CRE and AP-1 together drive the *Per* gene transcription (Narishige et al., 2014; Jagannath et al., 2021), which in turn participates in the transcriptional feedback loops that regulate circadian rhythms (Figure 3).

In addition, caffeine affects the release of neurotransmitters, such as γ-aminobutyric acid, dopamine, glutamate, acetylcholine, norepinephrine, and serotonin, between synaptic neurons in almost all brain areas by blocking the adenosine receptors (Nehlig et al., 1992; Cappelletti et al., 2015; Yang et al., 2021) (Figure 3), thereby significantly influencing the sleep-wake rhythms (Kumar and Lipshultz, 2019).

## 7 The effects of caffeine on circadian rhythms in preterm infants

Caffeine is widely prescribed to treat or prevent the AOP (Eichenwald, 2020; van Dam et al., 2020) and has recently been attempted to prevent the encephalopathy (Williamson et al., 2021; Yang et al., 2021) for preterm neonates in the NICU. Therefore, studies on the caffeine treatment in preterm infants mainly focus on the respiratory and neurodevelopmental outcomes (Schmidt et al., 2006; Schmidt et al., 2007), while less attention has been paid to its effects on their circadian rhythms.

In fact, the ultradian or irregular circadian rhythms due to the neurodevelopmental immaturity of preterm infants with different GA during the early postnatal life (Begum et al., 2006; Darnall et al., 2006) are more likely to mask caffeine’s effects. Moreover, preterm infants with different PNA and/or PMA experience different circadian characteristics (Mirmiran et al., 2003a; Darnall et al., 2006), so whether the response to caffeine therapy are partly related to the maturation of the circadian system in preterm infants remains to be explored.

Thus, relevant advances are summarized here to delineate those effects of caffeine on the circadian rhythms in preterm infants. Besides, theophylline and aminophylline, another two methylxanthines and fully metabolized in the body to produce the main metabolite caffeine (Bory et al., 1979; Pacifici, 2014), are also commonly used in the treatment of AOP (Henderson-Smart and De Paoli, 2010; Henderson-Smart and Steer, 2010; Eichenwald et al., 2016). The real effects of theophylline and aminophylline are thus thought to be related to caffeine in nature (Bory et al., 1979). Collectively, studies involving the effects of caffeine, theophylline, and aminophylline on the circadian rhythms in preterm infants are summarized in Tables 5, 6 and described as follows:

**TABLE 5:** Studies about the effects of methylxanthine on sleep-wake rhythms in preterm infants.

Studies	Subjects	Treatments	Methods of evaluation	Main findings
Seppä-Moilanen et al. (2021)	21 preterm infants (GA: 28.4–33.6 weeks)	Caffeine citrate (loading: 20 mg/kg; maintenance: 5 mg/kg/day)	Polysomnography	• Caffeine do not affect the sleep-arousal characteristics of preterm infants on the second day of treatment
Koch et al. (2020)	52 preterm infants (GA: 29.9 ± 1.96 weeks) *vs.* 12 preterm infants (GA: 33.4 ± 1.75 weeks)	Caffeine citrate (loading: 20 mg/kg; maintenance: 5–10 mg/kg/day) *vs.* no-caffeine	Videographic recordings	• In caffeine cohort with GA ≥ 28 weeks: AS↓ and wakefulness↑ as caffeine concentrations and PNA increased over the first 5 days of life
				• In caffeine cohort with GA < 28 weeks: no clear caffeine effects on sleep-wake behavior
				• In no-caffeine cohort: no PNA effects on sleep-wake behavior
Hassanein et al. (2015)	20 preterm infants (GA: 31.70 ± 1.16 weeks)	Caffeine citrate (loading: 20 mg/kg)	aEEG recordings	• A loading dose of caffeine leads to AS↓, QS↓, drowsiness↓, quite alert↑, active alert↑, crying↑
Lee et al. (2010)	35 preterm infants (GA: 24.9–31.9 weeks)	Aminophylline (loading: 5 mg/kg; maintenance: 1.5 mg/kg/8 h)	aEEG recordings	• The sleep-wake cycling is more prominent in preterm infants receiving aminophylline at 34–36 weeks PMA
				• Aminophylline use is associated with the appearance of sleep-wake cycling in preterm infants
Hayes et al. (2007)	14 preterm infants (GA: 28.6 ± 2.3 weeks) *vs.* 13 preterm infants (GA: 30.3 ± 1.5 weeks) *vs.* 10 preterm infants (GA: 32.4 ± 1.5 weeks)	Caffeine *vs.* theophylline *vs.* untreated control	Videographic recordings; Actigraphy	• Methylxanthine duration is associated with: AS↓, wakefulness↑, sleep-related movements↑
				• Methylxanthine *vs.* untreated: arousal rate↓, wakefulness↓, sleep-related movement↓ at night (from 24:00 to 05:00)
Chardon et al. (2004)	11 preterm infants (GA: 31.1 ± 1.8 weeks) *vs.* 11 preterm infants (GA: 30.3 ± 2.0 weeks)	Caffeine citrate (4.0 ± 0.5 mg/kg/day) *vs.* no-caffeine	Actigraphy; EEG; Eye movement monitors; Visual observations	• Caffeine has no significant effects on the TST, AS, QS, and IS for preterm infants during the inter-feeding intervals (2–3 h)
Curzi-Dascalova et al. (2002)	10 preterm infants (GA: 32.6 ± 0.21 weeks) *vs.* 5 preterm infants (GA: 32.7 ± 0.3 weeks)	Caffeine citrate (loading: 20 mg/kg; maintenance: 5 mg/kg/day) *vs.* no-caffeine	Polysomnography	• Caffeine has no significant effects on the AS, QS, IS, wakefulness, and state transitions for preterm infants during daytime (from 09:00 to 19:00) between 33 and 34 weeks PMA
Thoman et al. (1985)	4 preterm infants (GA: 28–30 weeks) *vs.* 5 preterm infants (GA: 29–35 weeks) *vs.* 28 term infants (GA: 37–42 weeks)	Theophylline *vs.* no-theophylline *vs.* untreated control	Direct behavioral observations	• Theophylline *vs.* no-theophylline for preterm infants at 2–5 weeks post-term: waking activity↑, alert↑, drowse or transition↑, AS↓
				• Theophylline preterm *vs.* untreated term infants: waking activity↑, alert↑, drowse or transition↑, AS↓, QS↓
Gabriel et al. (1978)	6 preterm infants (GA: 30.4–32.9 weeks)	Aminophylline (loading: 5.5 mg/kg; maintenance: 1.1 mg/kg/8 h)	Polysomnography	• Sleep cycles of AS, QS, and IS are unaffected during short-term theophylline treatment and after drug withdrawal
Dietrich et al. (1978)	9 preterm infants (GA: 26–32 weeks)	Aminophylline (loading: 5.8 mg/kg; maintenance: 1.4 mg/kg/8 h)	Direct behavioral observations; EEG	• During theophylline therapy *vs*. before theophylline therapy: AS↑, QS↓, IS↓, wakefulness↑

Abbreviations: aEEG, amplitude-integrated electroencephalography; AS, active sleep; EEG, electroencephalography; GA, gestational age; IS, indeterminate sleep; PMA, postmenstrual age; PNA, postnatal age; QS, quiet sleep; TST, total sleep time.

**TABLE 6:** Studies about the effects of methylxanthine on cardiorespiratory rhythms in preterm infants.

Studies	Subjects	Treatments	Methods of evaluation	Main findings
Seppä-Moilanen et al. (2021)	21 preterm infants (GA: 28.4–33.6 weeks)	Caffeine citrate (loading: 20 mg/kg; maintenance: 5 mg/kg/day)	Polysomnography	• Caffeine leads to SpO_2_↑, while HRV not changed on the second day of treatment
Williams et al. (2020)	32 preterm infants (GA: 27.27–31.49 weeks)	Caffeine citrate (loading: 20 mg/kg)	EMG	• A loading dose of caffeine leads to RR↑
Shivakumar et al. (2019)	185 preterm infants (GA: 29.5 ± 1.6 weeks)	Caffeine citrate (loading: 20 mg/kg; maintenance: 5 mg/kg/day) *vs.* aminophylline (loading: 5 mg/kg; maintenance: 1.5 mg/kg/8 h)	Echocardiography	• Aminophylline leads to HR↑, while caffeine has no significant increase in HR after 48 h of continued therapy compared with pretreatment values
Huvanandana et al. (2019)	40 preterm infants (GA: 23.6–33.3 weeks)	Caffeine base (loading: 10 mg/kg)	Intra-arterial blood pressure monitor; ECG	• A loading dose of caffeine leads to mean arterial pressure variability↑, pulse pressure variability↑, HRV↓
Dix et al. (2018)	34 preterm infants (GA: 28.8 ± 2.1 weeks)	Caffeine base (loading: 10 mg/kg)	Physiological parameter monitor	• A loading dose of caffeine leads to HR↑ and MABP↑ over time, while RR and SaO_2_ not changed
Dekker et al. (2017)	13 preterm infants (GA: 26–28 weeks) *vs.* 10 preterm infants (GA: 27–29 weeks)	Caffeine base (loading: 10 mg/kg)	Pulse oximeter	• A loading dose of caffeine leads to HR↑, while RR and SpO_2_ not changed
Parikka et al. (2015)	17 preterm infants (GA: 23.7–31.9 weeks)	Caffeine citrate (loading: 20 mg/kg)	Pulse oximeter	• A loading dose of caffeine leads to HR↑, while RR not changed
Hassanein et al. (2015)	20 preterm infants (GA: 31.70 ± 1.16 weeks)	Caffeine citrate (loading: 20 mg/kg)	Continuous cardiovascular and respiratory monitoring	• A loading dose of caffeine leads to HR↑, MABP↑, SpO_2_↑
Ulanovsky et al. (2014)	21 preterm infants (GA: 30.3 ± 2.5 weeks)	Caffeine citrate (loading: 15–20 mg/kg; maintenance: 5–10 mg/kg/day)	Cardiac monitor	• A loading dose of caffeine has no significant effects on HRV
Supcun et al. (2010)	51 preterm infants (GA: 24–33 weeks)	Caffeine base (loading: 10 mg/kg)	Cardiac monitor	• A loading dose of caffeine leads to MABP↑, while HR and SaO_2_ not changed
Soloveychik et al. (2009)	43 preterm infants (GA: 27.62 ± 2.94 weeks)	Caffeine citrate (5, 10, 20 mg/kg)	Continuous cardiovascular monitoring	• A dose of caffeine leads to BP↑, HR↑
Hoecker et al. (2006)	16 preterm infants (GA: 24–33 weeks)	Caffeine citrate (loading: 25 mg/kg/4 h; maintenance: 10 mg/kg/day)	Continuous cardiovascular and respiratory monitoring	• Two divided loading dose of caffeine lead to HR↑, diastolic BP↑, while RR not changed
von Poblotzki et al. (2003)	16 preterm infants (GA: 24.0–29.5 weeks)	Theophylline (5 mg/kg)	Continuous cardiorespiratory monitoring	• A dose of theophylline leads to HR↑, while RR and SpO_2_ not changed
Hoecker et al. (2002)	16 preterm infants (GA: 31 ± 1.2 weeks)	Caffeine base (loading: 25 mg/kg; maintenance: 5 mg/kg/day)	Continuous cardiorespiratory monitoring	• A loading dose of caffeine has no significant effects on BP and HR
Bauer et al. (2001)	18 preterm infants (GA: 28–33 weeks)	Caffeine citrate (loading: 10 mg/kg; maintenance: 5 mg/kg/day)	Continuous cardiorespiratory monitoring	• The RR, HR, and SaO_2_ are not significant changed at 48 h after caffeine treatment
Dani et al. (2000)	20 preterm infants (GA: 30.4 ± 3.0 weeks)	Caffeine citrate (loading: 10 mg/kg; maintenance: 2.5 mg/kg/day) *vs.* aminophylline (loading: 5 mg/kg; maintenance: 1.25 mg/kg/12 h)	Pulse oximeter; Continuous cardiorespiratory monitoring	• The HR, MABP, and SaO_2_ are not significant changed after caffeine or aminophylline treatment for at least 3 days
Carnielli et al. (2000)	18 preterm infants (GA: 32.7 ± 1.1 weeks)	Aminophylline (loading: 5 mg/kg; maintenance: 1.25 mg/kg/12 h)	Continuous cardiorespiratory monitoring	• A loading dose of theophylline leads to HR↑, RR↑
Govan et al. (1995)	20 preterm infants (GA: 28.0 ± 2.0 weeks)	Aminophylline (loading: 6 mg/kg)	Pulsed Doppler; Intra-arterial blood pressure monitor	• A loading dose of theophylline leads to HR↑, while MABP not changed
Chang and Gray, (1994)	10 preterm infants (GA: 27–32 weeks)	Aminophylline (loading: 7.5 mg/kg)	Cardiorespiratory monitor	• A loading dose of theophylline leads to HR↑, while MABP not changed
Bucher et al. (1994)	13 preterm infants (GA: 26–34 weeks)	Aminophylline (loading: 6 mg/kg)	Pulse oximeter; ECG	• A loading dose of theophylline leads to HR↑, while SaO_2_ not changed
McDonnell et al. (1992)	10 preterm infants (GA: 23–31 weeks)	Aminophylline (loading: 6.2 mg/kg)	Pulse oximeter; Intra-arterial blood pressure monitor	• A loading dose of theophylline leads to HR↑, while MABP not changed
Pryds and Schneider, (1991)	16 preterm infants (GA: 25–34 weeks)	Aminophylline (loading: 10 mg/kg)	Intra-arterial blood pressure monitor	• A loading dose of theophylline has no significant effects on MABP
Walther et al. (1990)	10 preterm infants (GA: 29.6 ± 3.0 weeks)	Caffeine citrate (loading: 20 mg/kg; maintenance: 5 mg/kg/day)	ECG; Oscillometry	• Caffeine leads to MABP↑ during first 3 days treatment, while HR not changed
Saliba et al. (1989)	7 preterm infants (GA: 31.3 ± 2.0 weeks)	Caffeine citrate (20 mg/kg) or saline	ECG; Oscillometry	• A loading dose of caffeine leads to HR↑, while MABP not changed
Walther et al. (1986)	10 preterm infants (GA: 30.7 ± 0.8 weeks)	Aminophylline (loading: 6.8 mg/kg; maintenance: 2 mg/kg/8 h)	Pulsed Doppler; Echocardiography	• Theophylline leads to HR↑ during first 7 days treatment, while MABP not changed

Abbreviations: BP, blood pressure; bpm, beats per minute; ECG, electrocardiography; EMG, electromyography; GA, gestational age; HR, heart rate; HRV, heart rate variability; MABP, mean arterial blood pressure; PMA, postmenstrual age; PR, pulse rate; RR, respiratory rate; SaO_2_, arterial oxygen saturation; SpO_2_, pulse oximeter oxygen saturation.

### 7.1 The effects on sleep-wake rhythms

The well-studied effects of caffeine on sleep-wake rhythms in preterm infants are still limited as the sample sizes were small and the study designs were heterogeneous (Table 5). Some studies revealed that the sleep-wake patterns were not significantly changed after short-term treatment with caffeine or theophylline during short observation periods (Gabriel et al., 1978; Curzi-Dascalova et al., 2002; Chardon et al., 2004; Seppä-Moilanen et al., 2021).

However, some other studies observed significant effects of caffeine on the sleep-wake rhythms, although these effects were not entirely consistent (Dietrich et al., 1978; Thoman et al., 1985; Hayes et al., 2007; Hassanein et al., 2015; Koch et al., 2020). For example, Koch et al. (2020), found that the AS decreased while the wakefulness increased but QS unchanged as caffeine concentrations and the PNA increased over the first 5 days of life in preterm infants more than 28 weeks of GA, but no clear effects on the sleep-wake states were found in preterm infants less than 28 weeks of GA, and no such PNA effects were found in no-caffeine cohort. Hassanein et al. (2015) also detected significant decreases in the AS, QS, and drowsiness, while increases in the quite alert, active alert, and crying in preterm infants half an hour after caffeine administration. Similar methylxanthine-induced changes in the AS and wakefulness states were also observed in studies conducted by Hayes et al. (2007) and by Thoman et al. (1985). However, Dietrich et al. (1978) found the AS and wakefulness increased while the QS and IS decreased during theophylline therapy.

In addition, Lee et al. (2010) discovered that the appearance of sleep-wake cycling was associated with the aminophylline use and more prominent. However, in the prospective follow-up study of the CAP trial (Marcus et al., 2014), no significant differences in sleep states were found in preterm infants aged 5–12 years who had been treated with caffeine after birth compared with the placebo group, which possibly due to the apparent discrepancy in total recording and sleep time between the two groups.

This is also true for some animal studies. Denenberg et al. (1982) found that theophylline reduced the AS, while increased wakefulness, delayed the development of QS, and affected the intermediate states of sleep-wake and AS-QS transitions in newborn rabbits. Montandon et al. (2009) also discovered that the sleep time was reduced, sleep onset latency was increased, and non-REM sleep was fragmented in adult rats treated with caffeine compared to controls during the neonatal period.

Due to the heterogeneous designs and inconsistent results of the above studies, it is difficult to draw clear conclusions. Nonetheless, it can be summarized that caffeine affects the sleep patterns in preterm infants, especially the AS and wakefulness, and the effects might persist into the childhood and even the adulthood. If this hypothesis holds true, then the inhibition of adenosine receptors by caffeine would exactly explain the altered sleep-wake states in preterm infants, as the association between caffeine, adenosine, and sleep has been well documented in adults (Huang et al., 2011; Porkka-Heiskanen and Kalinchuk, 2011; Huang et al., 2014a; Urry and Landolt, 2015; Reichert et al., 2022). In addition, the alteration of sleep-wake patterns might be partially responsible for the caffeine-induced increase in cerebral cortical activity (Supcun et al., 2010; Hassanein et al., 2015) and decrease in apneic episodes (Dietrich et al., 1978; Montandon et al., 2009; Seppä-Moilanen et al., 2019; Seppä-Moilanen et al., 2021).

### 7.2 The effects on cardiorespiratory rhythms

Current studies have confirmed that caffeine acts both peripherally and centrally to stimulate respiration mainly via inhibiting the adenosine A_1_ and A_2A_ receptors (Abdel-Hady et al., 2015; Eichenwald et al., 2016; Dobson and Hunt, 2018). Caffeine activates the medullary respiratory center, improves sensitivity to carbon dioxide, increases respiratory muscle strength, enhances diaphragmatic contractility, and induces bronchodilation (Kassim et al., 2009; Parikka et al., 2015; Dekker et al., 2017; Sanchez-Solis et al., 2020; Williams et al., 2020), which synergistically cause the increased minute ventilation and oxygen consumption, while cause the decreased apnea, periodic breathing, and intermittent hypoxia (Seppä-Moilanen et al., 2021; Seppä-Moilanen et al., 2019; Dobson et al., 2017; Rhein et al., 2014; von Poblotzki et al., 2003; Bauer et al., 2001; Carnielli et al., 2000).

In addition, caffeine or theophylline therapy increases the cardiac output, stroke volume, and metabolic rate (Walther et al., 1986; Walther et al., 1990; Carnielli et al., 2000; Bauer et al., 2001; Soloveychik et al., 2009; Shivakumar et al., 2019), but decreases blood flow velocities in cerebral and intestinal arteries (Pryds and Schneider, 1991; McDonnell et al., 1992; Bucher et al., 1994; Chang and Gray, 1994; Govan et al., 1995; Lundstrøm et al., 1995; Lane et al., 1999; Hoecker et al., 2002; Hoecker et al., 2006; Dix et al., 2018; Hwang et al., 2018; Abdel Wahed et al., 2019) for preterm infants, which appeared to be related to the enhanced endothelial function through antagonism of adenosine receptors, inhibition of phosphodiesterase, and through promotion of intracellular calcium concentrations (Higashi, 2019). Although the clinical significance remains unclear, this reduced perfusion activity was a reminder that caffeine might have adverse effects on the developing brain and gastrointestinal tract (McDonnell et al., 1992; Lane et al., 1999; Hoecker et al., 2002; Hoecker et al., 2006; Atik et al., 2017; Abdel Wahed et al., 2019).

Unlike the cardiopulmonary system, the effects of caffeine on the cardiorespiratory rhythms in preterm infants have not been specifically studied. Nonetheless, the effects of caffeine on the heart rate, respiratory rate, blood pressure, and oxygen saturation have been examined. As summarized in Table 6, some studies found that a loading of caffeine or theophylline increases the heart rate (Hassanein et al., 2015; Dekker et al., 2017; Parikka et al., 2015; von Poblotzki et al., 2003; Carnielli et al., 2000; Soloveychik et al., 2009; Dix et al., 2018; Govan et al., 1995; Chang and Gray, 1994; Bucher et al., 1994; McDonnell et al., 1992; Saliba et al., 1989), blood pressure (Soloveychik et al., 2009; Supcun et al., 2010; Hassanein et al., 2015; Dix et al., 2018; Huvanandana et al., 2019), respiratory rate (Williams et al., 2020), and oxygen saturation (Hassanein et al., 2015), which were in line with those studies with multiple caffeine dosing (Walther et al., 1986; Walther et al., 1990; Hoecker et al., 2006; Shivakumar et al., 2019). Those findings reflected the complex effects, directly or indirectly like the enhanced autonomic nervous system responsiveness (Huvanandana et al., 2019), of caffeine on the cardiopulmonary system. However, several other studies did not find similar effects (Pryds and Schneider, 1991; Dani et al., 2000; Bauer et al., 2001; Hoecker et al., 2002; Ulanovsky et al., 2014).

Unfortunately, no research has touched this area yet in premature infants until now. It is worth mentioning that neonatal caffeine treatment upregulates adenosine receptors in cardiorespiratory related nuclei of the rat brain (Gaytan et al., 2006; Gaytan and Pasaro, 2012), and this effect persists into the adulthood (Bairam et al., 2009), which underscores the urgent to study the potential long-term effects of caffeine on the cardiorespiratory system in preterm infants (Montandon et al., 2008). In view of the complex and profound effects of caffeine in this field, systematic and in-depth research is still necessary.

### 7.3 The effects on other rhythms

Two studies recorded the body temperature of preterm infants and incubator temperature during short-term caffeine administration. Chardon et al. (2004) found that caffeine has no significant effect on the skin temperature and incubator temperature. However, Bauer et al. (2001) observed that a lower incubator temperature was sufficient to maintain a normal body temperature for preterm infants after caffeine treatment, which might be related to the increased metabolism caused by methylxanthines (Bucher et al., 1994; Carnielli et al., 2000; Bauer et al., 2001). However, the effects of caffeine on circadian body temperature rhythms have not been extensively studied. Similarly, although caffeine has been shown to affect melatonin (Wright et al., 1997; Wright et al., 2000; Burke et al., 2015) and cortisol (Lovallo et al., 2005; Rieth et al., 2016) rhythms in adults, these effects in premature infants still need to be addressed.

Collectively, the relevant research on the circadian rhythms in premature infants receiving caffeine therapy is still scarce. Although existing studies have suggested the possible effects of caffeine on the circadian rhythms, heterogeneity in study designs and inconsistency in conclusions weaken the power of those evidence. More research is needed in the future to confirm the effects of caffeine and the underlying mechanisms. The story should not end here.

## 8 Circadian-based caffeine therapeutic strategies for AOP: New possibility opens up

**FIGURE 4:**
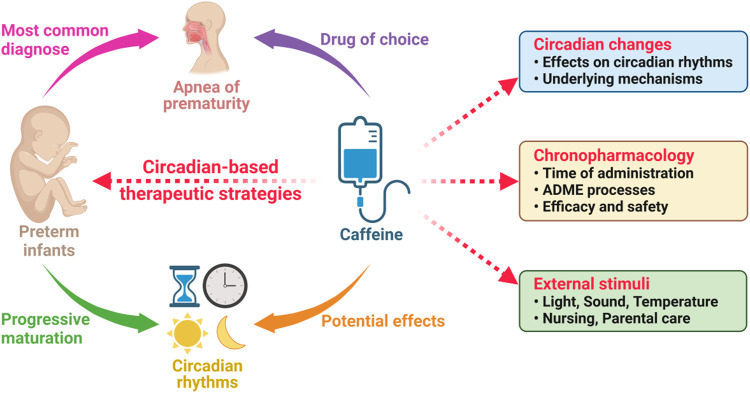
The circadian-based therapeutic strategies of caffeine in preterm infants with apnea of prematurity. ADME, absorption, distribution, metabolism, and excretion.

It is estimated that more than 15 million neonates are born preterm globally each year, and the preterm birth appears to be increasing in most countries (Vogel et al., 2018; Walani, 2020; Deng et al., 2021). Premature babies may have various problems like AOP. Unfortunately, the tough challenges are always there for the current AOP therapy, such as significant interindividual variability in the response to caffeine (Saroha and Patel, 2020; He et al., 2021). Intriguingly, one most recent study revealed that the *Clock* gene polymorphisms were significantly associated with the response to caffeine therapy in preterm infants (Guo et al., 2022). Although the molecular action mechanism through which there is a better response is unknown, these results show that the circadian rhythms might play a critical role in response to the therapy. In this way, a new possibility opens up in this area of research (Figure 4), and we tentatively propose three initiatives.

### 8.1 Considering the circadian changes

As discussed above, the efficacy of caffeine appeared to interact with the circadian rhythms in premature infants. Studies have demonstrated the significant effects and underlying mechanisms of caffeine in adults and in animals (Landolt, 2015), but it remains unclear whether the similar mechanisms also exist in those preterm infants. The effects of caffeine on the circadian rhythms, especially the sleep-wake rhythms, are advised to be considered into the strategy of the caffeine therapy (Figure 4).

In addition, studies have revealed that several circadian-related problems like sleep, breathing, and blood pressure in premature infants may persist into childhood and even adulthood (Weisman et al., 2011; Huang et al., 2014b; Sipola-Leppanen et al., 2015; Caravale et al., 2017; Durankus et al., 2020). Based on the existing evidence, it is feasible to propose that caffeine’s effects on circadian rhythms may ameliorate those problems and promote the maturation of circadian rhythms in preterm infants to the level of normal term infants.

### 8.2 Considering the chronopharmacology

The concept of chronopharmacology holds that the ADME processes and the sensitivity of a biological target to a drug are determined by the endogenous biological circadian oscillations (Ohdo et al., 2019; Bicker et al., 2020; Dong et al., 2020; Dobrek, 2021). Variable efficacy and safety profiles would be exhibited for many drugs if they are administered at different times of the day (Dallmann et al., 2016; Cederroth et al., 2019; Nahmias and Androulakis, 2021). For preterm infants, interestingly, several circadian-related gene polymorphisms were found to be significantly associated with the response to caffeine therapy for AOP (Guo et al., 2022). It remains unclear whether caffeine administrated at different times of the day would cause changes in the ADME processes and the therapeutic effects, but it really opens a possibility to applicate the chronopharmacology in the NICU.

Although less research is currently available, there are rare but thought-provoking reports that arouse our strong interests (Smolensky et al., 1987; Pelissier-Alicot et al., 2002), which will lead us into a wonderland in the future. For examples, Pelissier-Alicot et al. (2002) found that the pharmacokinetic profiles of caffeine in rats, such as the clearance, volume of distribution, and area under the plasma concentration-time curve (AUC), depended strongly on the time of day of administration, while the daily rhythmicity of heart rate, body temperature, and locomotor activity in rats also changed with the dosing time of caffeine. Similarly, Smolensky et al. (1987) demonstrated that the pharmacokinetic profiles and therapeutic effects of theophylline in asthmatic children varied with the dosing time. These findings attract us that the circadian rhythms might play a critical role in the ADME processes as well as the efficacy and safety of caffeine therapy in preterm infants.

Currently, caffeine is now commonly administered once daily in preterm infants (Long et al., 2021). The question is whether we are willing to make positive attempts to tailor the dosing time according to the principles of chronopharmacology. If the significant association between circadian-related gene polymorphisms and response to caffeine therapy in preterm infants (Guo et al., 2022) were true and phenotypically manifested, then the administration at different time points of the day is more likely to witness those potentially altered pharmacokinetics of and clinical response to caffeine.

Maintaining normal circadian rhythms are necessary to stay health. Essentially, caffeine interferes with these rhythms to a certain extent, and its arousal effects are very important for the AOP management among various pharmacological mechanisms. Therefore, whether to apply caffeine in accordance with the circadian rhythms to maintain the stabilities of these rhythms as much as possible, or to subtly counteract these rhythms to amplify its arousal effect and achieve a better therapeutic effect, all these aspects deserve our in-depth consideration (Figure 4).

### 8.3 Considering the other external stimuli

If the homeostasis of circadian rhythms were necessary for health, then correcting the possible adverse effects due to preterm birth is a matter that needs to be taken seriously in the NICU, including the effects on the treatment drugs being used. As discussed above, several external stimuli or known as zeitgebers, such as light, sound, temperature, nursing, and parental care, *etc.*, play important roles in the maturation of circadian rhythms. Cycled light (Abraham et al., 2006; Ohta et al., 2006; Bode et al., 2011), music therapy (Arnon et al., 2006; Loewy et al., 2013), appropriate incubator temperature (Tourneux et al., 2008), comfortable nursing (Collins et al., 2015; Lan et al., 2018), and even the adequate parental care (Löhr and Siegmund, 1999; Nishihara et al., 2002; Park et al., 2020) are helpful for the development and maturation of the circadian rhythms in neonates.

Therefore, the beneficial effects of those external stimuli on the circadian rhythms for premature infants cannot be ignored in the NICU, taking the application of caffeine to manage the AOP for example (Figure 4). Coordinating all treatment strategies with the principles of circadian rhythms will be a constructive attempt to improve the disease management and care for premature infants. Assuredly, we have to admit that only rare evidence is available currently, and the realization of the therapeutic strategies cannot be achieved overnight. However, any kind of discussions, attempts, and efforts in this field should well be encouraged in the future.

## 9 Conclusion

Due to the tough challenges and potential role of circadian rhythms in the response to current caffeine therapy for the AOP management, a comprehensive review was conducted here. Studies have revealed that the human circadian system begins to form in early pregnancy, receives the maternal circadian signals through the placenta before birth, and progressively matures under the influence of the external cues and the mother after birth. Preterm infants experience the ultradian or irregular rhythms during the early postnatal life, which are progressively developed into circadian rhythms as the maturation of neurodevelopment. Caffeine alters the circadian rhythms in humans and animals, and its promising role in preterm infants has also been revealed. The proposed novel circadian-based therapeutic strategies could open new possibilities in the clinical practice to promote the precision caffeine therapy. Arguably, as studies going on, it is believed that in the near future, these initiatives will remain powerful approaches to enhance our biological understanding of the relationship between preterm infants, circadian rhythms, and caffeine therapy.
